# Flavor changes of Yunnan large-leaf cultivar white tea during different aging periods

**DOI:** 10.1016/j.fochx.2025.102846

**Published:** 2025-07-29

**Authors:** Caibi Zhou, Yongshi Chen, Mengling Chen, Mei Xu, Dongwei Zhao, Long Huang, Wenpin Chen

**Affiliations:** a College of Horticulture, South China Agricultural University/Guangdong South China Feature Tea Engineering Research Center, 510642 Guangzhou, China; b College of Forestry/Ancient Tea Tree Research Center, Southwest Forestry University, Kunming 650224, China; c University of Saskatchewan, 573440 Saskatoon, Canada; d Yumingju Technology Co., Ltd, 528400 Zhongshan, China

**Keywords:** White tea, Storage time, Sensory evaluation, Non-volatile components, Volatile components, Electronic nose, Safety evaluation

## Abstract

Yunnan large-leaf white tea gains popularity for its unique flavor attributable to the local tea varieties and environments. But how aging crucially enhances its quality and flavor remains unclear. Here, flavor change and safety quality of “Qinghuan” (QH) stored for different periods were studied, revealing that older teas developed sweeter, smoother, and more balanced flavors, with a stable and pleasant aroma. In total, 32 key non-volatiles (e.g., rutin, (−)-epigallocatechin gallate, p-coumaric acid) were screened by principal component analysis and significant changes were observed in volatiles (alcohols, aldehydes, terpenes) over time. Additionally, aflatoxins in QH teas remained undetected, irrespective of aging time; and oral median lethal doses of 2023 and 2017 QH teas were 17.17 g/kg·bw and 15.09 g/kg·bw, indicating that QH tea has a high safety quality for drinking over the aging time. Overall, QH tea's flavor and safety quality increased with storage time, providing evidence of the temporal value for collecting white tea.

## Highlights

• Storage duration significantly impacted the color, aroma, and taste of white tea*.*
• *α*-ionone, *β*-cyclocitral, tetradecane, and dehydro-*β*-ionone were the key VOCs in QH.
• The one-year and seven-year aged white teas were classified as practically non-toxic.
• Caffeine and ellagic acid affected liquor color and flavor of QH during storage.

## 1 Introduction

White tea, as one of the six major categories of tea, is a lightly fermented tea primarily produced in Fujian and Yunnan provinces (Zhou et al., 2023). It is favored for its fresh, smooth, and pure aroma, as well as its sweet and refreshing taste. Tea cultivars, harvesting standards, and storage time are key factors influencing the flavor and quality of white tea. “Yunnan large-leaf tea cultivars” refers to a general term for various large-leaf tea cultivars of arbors and small deciduous trees that are distributed across the tea regions of Yunnan Province. Yunnan large-leaf white tea (YNWT) is produced from fresh tea leaves through a series of specific processes, including withering, sun-drying, refining, steaming or non-steaming, followed by drying. YNWT is rich in phenolic acids, flavonoid glycosides (e.g., kaempferol-3-galactoside and quercetin-3-glucoside), organic acids, lipids, and epicatechins, dimericcatechins (Gao et al., 2022). In addition, this variety is abundant in *R*-linalool, *R*-linprim, and methyl salicylate, primarily presented sweet, plum-like, and vibrant floral aromas. Fujian white teas contained an abundant of linalool, *α*-citral, and *R*/*S*-dihydroactinidiolide, characterized by pekoe, floral fragrance, and refreshing aroma (Yan et al., 2024). White tea has been reported to be rich in caffeine and polysaccharides, with particularly higher concentrations of polyphenols and amino acids compared to other tea varieties. Consequently, white tea is considered to possess several health benefits such as anti-diabetic, antioxidant, anti-inflammatory, and anti-cancer properties, as well as the potential to alleviate nervous tension (Jacobson et al., 2022).

It is said that “white tea is one year for tea, three years for medicine, and seven years for treasure”, indicating that the chemical components, biological function and flavor quality of white tea improve during the aging process. The aging process in white tea is characterized by transformation of various compounds, which significantly impacts its flavor, aroma, taste, and color. As aging progresses, dry-tea's color, infusions, and residues gradually deepen over time. Additionally, the aging process imparts a delightful aroma to white tea and enhances its overall flavor profile (Fan et al., 2021). The flavor of tea is predominantly determined by essential compounds associated with its flavor, including tea polyphenols, free amino acids, and caffeine. During the aging process of white tea, the levels of total flavonoids, thearubigins (TRs), soluble sugars, and theabrownines (TBs) exhibited an increase, whereas total polyphenols demonstrated a gradual decline (Zhao, Chen, et al., 2022). Theogallin, *γ*-aminobutyric acid (GABA), caffeine, (−)-epigallocatechin gallate (EGCG), and (−)-epicatechin gallate (ECG) have been identified as the principal taste compounds in white tea (Yang et al., 2018). Amino acid was responsible for the development of umami flavor. Asparagine, theanine, and aspartic acid exhibited a positive correlation with the umami taste in white tea, whereas the content of total amino acid demonstrated a negative correlation over time (Fan et al., 2021). Although, several amino acid monomers undergo distinct transformation during the aging process of tea. For instance, the concentrations of glutamic acid and threonine in white tea were observed to increase during short-term aging as a result of protein hydrolysis, whereas pyroglutamic acid, a cyclic derivative of glutamic acid, increased after long-term aging (Xie et al., 2019). Flavan-3-ols, theasinensins, procyanidin B3, and theobromine exhibited a positive correlation with higher bitterness and astringency. Various chemical reactions including oxidation, complexation, and hydrolysis caused a decrease in the concentrations of alkaloids and tea polyphenols during the aging process. In addition, soluble sugars, recognized as the primary substances responsible for sweetness, demonstrated an increasing trend as a result of protein hydrolysis, polyphenol oxidation, and the activation of *α*-glucosidase and amylase (Zhou et al., 2022). In summary, the aging process of white tea reduced the levels of polyphenols and amino acids, thereby diminishing its umami, bitterness, and astringency; in contrast, the increased levels of soluble sugars, flavonoids, theaflavins (TFs), and gallic acid (GA) correspondingly enhance the sweetness, thickness, smoothness, and acidity; additionally, the transformation of volatiles revealed more pronounced herbal and sweet flavors (Zhou et al., 2023).

Aroma substances undergo complicated chemical transformations during the aging process of white tea. The aromatic characteristics of white tea are distinguished by fresh, fruity, sweet, and floral notes, which are primarily influenced by volatiles such as hydrocarbons, aldehydes, alcohols, ketones, and esters. Among these, alcohols serve as the predominant aromatic components in white tea (Wang, Shi, et al., 2022). Particularly, the aroma of white tea is significantly affected by storage time. The concentration of alcohols was highest during the storage period of 1 to 5 years, while the concentration of esters reached its peak during the storage period of 7 to 13 years, and the concentration of aldehydes was maximized after a storage duration of 16 years (Wang, Fu, et al., 2022). Additionally, methyl salicylate, cedrol, and linalool II have been identified as characteristic compounds of new white tea, whereas *β*-damascenone and jasmine ketone were specific to aged white tea (Ma et al., 2023). The most abundant volatiles present in non-stored white tea are alcohols (e.g., geraniol, linalool, and their oxides), aldehydes (e.g., benzaldehyde and phenylacetaldehyde), and ketones (e.g., trans-*β*-ionone and *α*-ionone) (Chen et al., 2020). However, as the storage duration increases, the total aroma of white tea showed a decreasing trend; especially volatile compounds of alcohols (e.g., geraniol, linalool, phenylethanol, and nerolidol) were important sources of aroma (Wang, Wang, et al., 2022); whereas the decrease in their content leaded to a reduction in the fresh aroma of white tea, and a significant improvement in sweetness and herbal aroma (Qi et al., 2018). Although the variation in aromatic substances may be inconsistent, the overall fragrance of white tea remains harmonious. The aroma gradually transitions from fresh, sweet, fruity, and floral notes to aged, woody, herbal, orange, and peony scents (Wang, Wang, et al., 2022). Furthermore, advancements in technology have facilitated the widespread application of electronic nose (*E*-nose) and gas chromatography–mass spectrometry, which provide objective criteria for the sensory evaluation of tea aroma.

During the aging duration of white tea, the flavor and quality of tea undergo significant changes due to the transformation of chemical substances, particularly attracting attention for its health benefits. White tea was abundant in flavonoids with antioxidative and anti-inflammatory properties that could activate Nrf2/HO-1 pathway, enhance Trx expression, and directly eliminate reactive oxygen species (Berilli et al., 2022), thereby alleviating oxidative stress (OS). As aging time progressed, the particle size of white tea nanoparticles decreased gradually, uncovered the anti-aging mystery of white tea in another thread. The long-term consumption of white tea has been shown to protect tissues from acute oxidative stress; however, it did not mitigate the effects of chronic oxidative agents such as aging (Cristóbal et al., 2018), but antioxidant activity decreased with the prolongation of aging time. The inhibitory effects on the key enzymes s associated with type II diabetes, especially *α*-amylase and *α*-glucosidasewere, observed in the similar trend (Xu et al., 2019). In addition, white tea can activate phosphatidylinositol-3-kinase/protein kinase B (PI3K/Akt) pathway, regulate glucose and lipid metabolism, and prevent insulin resistance (IR) in mice (Xia et al., 2020). White tea served as an effective anti-inflammatory agent by enhancing the expression of the beneficial anti-inflammatory protein cytokine adiponectin (Hamdy et al., 2022), consequently relieving inflammation symptoms. The EGCG and ECG present in white tea acted as a crucial role in regulating cholesterol genes, which effectively modulated the signaling pathways involved in lipid metabolism, resulting in the downregulation of low density lipoprotein cholesterol (LDL-C) and triglyceride levels (Luo et al., 2020).

With the health benefits of white tea being recognized by consumers, as well as its more harmonious taste and enhanced effects after proper storage, it has gained collectible value. However, there is a lack of systematic research regarding the variations in flavor quality and drinking safety of white tea after long-term storage. In this study, white tea produced from the one-bud and three-leaf raw material of Yunnan large-leaf tea variety was analyzed for changes in sensory flavor, quality components, and drinking safety during the aging process. This study aims to provide insights for the flavor analysis and quality assessment of aged Yunnan large-leaf white tea, as well as for the aging technology of white tea.

## 2 Materials and methods

### 2.1 Samples and chemicals

Fresh tea leaves were harvested from Mengku tea garden (N 99.085487, S 23.559635) of Hongrun Lai Tea Co., Ltd. (Kunming City, China). All samples named Qinghuan (QH) underwent standardized processing procedures (Standardization Administration of China, 2016) in April of 2017, 2019, 2021, and 2023, denoted as 17QH, 19QH, 21QH, and 23QH, respectively. Briefly, fresh leaves piled with a thickness of 2–3 cm were withered for 45 h at an average temperature of 24 °C until the moisture content of leaves reached 20 %, followed by hot-air drying at 80 °C to stabilize the leaves at 8 % moisture content. All QH samples, totaling 12 tea samples with each weighing 50 kg, were hermetically sealed in aluminum foil bags and stored at room temperature in Guangzhou (yearly average 24 °C). Then all samples were collected randomly in May 2023, and stored at −80 °C for further analysis. 17QH and 23QH were employed as the experimental material of acute toxicity test.

Four-week-old healthy male and female Kunming mice with a body weight of 18 ± 2 g (Grade SPF, certificate No. SCXK 2020–0051) were purchased from Baishitong Biotechnology Co., Ltd. (Zhuhai, China). The mice were housed in an environment characterized by a 12-h light/dark cycle with a temperature of 20–25 °C and a relative humidity of 70–90 %.

Methanol, indenone, folinol reagent, anhydrous sodium carbonate, stannous chloride, disodium hydrogen phosphate dodecahydrate, anthrone, potassium dihydrogen phosphate, and acetonitrile were purchased from Thermo Fisher Scientific (Waltham, MA, USA). EGCG, ECG, epigallocatechin (EGC), gallo­catechin gallate (GCG), epicatechin (EC), GA, catechin (C), and caffeine were obtained from Yuanye Bio-Technology Co., Ltd. (Shanghai, China). *D*-(−) quinic acid, 3.4-dihydroxyphenylpropionic acid, vitexin, anthocyanins, caffeic acid, genistein, kaempferol, isoferulic acid, p-hydroxybenzoic acid, quercetin, p-coumaric acid, isovitexin, isoquercetin, quercetin, naringin, isorhamnetin, daidzein, 3,4-dihydroxyphenylacetic acid, proanthocyanidin B1, catechol, cryptochlorogenic acid, tannic acid, vitex-2-orhamnoside, sinapine, eugenol, phloroglucinol, gallic acid, protocatechuic acid, chlorogenic acid, anthocyanins B2, syringic acid, vanillin, rutin, hyperoside, ferulic acid, genistein, benzoic acid, cinnamic acid, vanillic acid, and kaempferol-3-*O*-glucoside were obtained from Sigma Aldrich Co., Ltd. (Beijing, China). Ethyl decanoate was purchased from Macklin Co., Ltd. (Beijing, China). C8-C40 alkane mixed standard was obtained from TMstandard Co., Ltd. (Beijing, China).

### 2.2 Sensory evaluation and chromaticity value determination

Sensory evaluation was carried out according to the Chinese national evaluation standard procedure for tea (GB/T 23776–2018). Briefly, 100 g tea samples were taken from aluminum foil bags and placed in a sample tray for appearance evaluation. Then, 3 g of tea sample was brewed with a ratio of 1:50 of tea to fresh boiling water for 5 min. After that, the infusion was evaluated for its color, taste, aroma, and the condition of the brewed leaves. Each attribute was evaluated and accompanied by professional commentary. The sensory panel consisted of 12 professional tea evaluators (six men and six women). Human sensory evaluation does not require ethical permission, and all panelists gave consent to participate in this research. All samples were presented randomly. The chromaticity value of liquor color, included *L*, *a*, and *b* values was determined using CM-5 spectrophotometer.

### 2.3 Analysis of non-volatile metabolites

Twelve tea leaf samples were processed using a high-speed multifunctional crusher and subsequently sifted through a 40-mesh screen for future use. Water content in tea, tea water extract, tea polyphenol, and total free amino acid were determined in accordance with Chinese national standards GB/T 31740.2–2015, GB/T 8305–2013, GB/T 8313–2018, and GB/T 8314–2013, respectively.

The concentrations of caffeine, GA, and catechins (GC, EGC, C, EC, EGCG, GCG, ECG, CG) were quantified using a Waters Model 2695 HPLC system equipped with a C18 column (Phenomenex, 4.6 × 250 mm, 5 μm). The mobile phase A consisted of 0.1 % formic acid, while mobile phase B comprised acetonitrile. The gradient of mobile phase B was as follows: 8 % for 0–5 min, increasing from 8 % to 25 % at 6–14 min, decreasing from 25 % to 8 % at 15–19 min, and maintaining at 8 % from 20 to 27 min. Flow rate: 0.9 mL/min, injection volume: 10 μL, column temperature: 25 °C, and ultraviolet (UV) detection at 280 nm. The standards of catechin and caffeine were employed to generate standard curves for quantification.

The concentration of flavonoid glycosides was quantified using UPLC-Q-Exactive Orbitrap-MS/MS system equipped with a C18 column (Agilent Poroshell HPH, 2.1 × 150 mm, 4 μm). All compounds were analyzed in triplicate to ensure the reliability and validity of the results. Quantitative analysis of flavonoid glycosides in 12 samples was performed with external analytical curves.

### 2.4 Analysis of volatile metabolites

The aroma characteristics of 12 samples were assessed using the PEN3 nose (ATRSENS, Germany), which was equipped with 10 metal oxide sensors (W1C/S1, W5S/S2, W3C/S3, W6S/S4, W5C/S5, W1S/S6, W1W/S7, W2S/S8, W2W/S9, W3S/S10) and an HP-5 headspace auto sampler. In brief, 1 g of tea sample was ground using liquid nitrogen and subsequently infused with 10 mL of boiling water in a 50-mL headspace vial. The vial was maintained at 45 °C for 30 min while stirring at 500 rpm, after which the sample was transferred into the *E*-nose injector. For testing conditions: The measurement time, zeroing time, pre sampling time, and rinse time were set to 60 s, 10 s, 5 s, and 100 s, respectively; the injection flow rate was set as 300 mL/min; balance time was 45 s. Data were analyzed within the response interval of 57–59 s. Airsens Winmuster software was employed to analyze the collected data, from which the stable response values of each sensor were extracted to serve as the E-nose data for each sample. The array of electronic nose sensors and their performance characteristics are presented in Table S1.

Volatile compounds were extracted from each sample using a modified headspace solid phase microextraction (HP-5 MS) method and subsequently analyzed with a gas chromatography coupled with mass spectrometry (GC–MS) system (7089B-5977 A, Agilent, Japan). Briefly, 1 g of sample was brewed with 5 mL of boiling water in a 40-mL headspace vial. Subsequently, 10 μL (1 μL/mL) of ethyl decanoate was added as an internal standard (IS) and the vial was sealed immediately. After securing the 50/30 μm DVB/CAR/PDMS extraction head (SUPERCO, USA) to the upper end, the headspace vial was maintained at 60 °C for 40 min to facilitate the absorption of volatile gases. Detection conditions were established as follows: capillary column, HP-5 MS (30 m × 0.25 mm × 0.25 μm); inlet temperature set at 250 °C; carrier gas consisting of high-purity helium (purity ≥99.99 %); column flow rate maintained at 1.0 mL/min; heating program commencing at an initial temperature of 40 °C, increasing to 150 °C at 3 °C/min and holding that temperature for 2 min, followed by an increase to 180 °C at 5 °C/min and holding for 2 min, and finally reaching 230 °C at a rate of 10 °C/min; injector mode configured as splitless. Ion source operated with an electron energy of 70 eV at an ion source temperature of 230 °C and a fourth-level rod temperature of 150 °C. The mass scanning range was set to 30–400 *m*/*z*. Volatiles were characterized using the mass spectral database and retention index (RI) derived from N-alkanes ranging from C8 to C40 combined with standard, as provided by the National Institute of Standards and Technology (NIST 14.0, Gaithersburg, MD, USA). The semi-quantitative method utilizing ethyl decanoate as an internal standard was employed for the relative quantification of volatile compounds (Luo et al., 2025; Wang, Wang, et al., 2022). Due to the equilibrium-based nature of SPME, the reported volatile compound concentrations are semi-quantitative, representing relative abundances normalized to the internal standard (ethyl decanoate).

The relative concentration of identified volatile compounds was calculated using the following equation: Relative concentration (μg/kg) = (Peak area of target/Peak area of IS).

### 2.5 Aflatoxins determination and safety evaluation of QH

Aflatoxins (AFs) in QH teas were detected using UPLC-Q-Exactive Orbitrap-MS/MS system equipped with a C18 column (Agilent Poroshell HPH, 2.1 × 150 mm, 4 μm) in accordance with local standards in Anhui Province (DB34/T 4268—2022, Determination of aflatoxin B and G groups in fermented tea).

Median lethal dose (LD_50_) is commonly utilized as an indicator for assessing the acute toxicity of food substances and chemicals. The acute oral toxicity study of Yunnan large-leaf white tea was conducted according to above guidelines. Mice were acclimated for three days prior to the experiments, during which they had unrestricted access to standard diets and distilled water, then those qualified mice were weighed and labeled with ear and tail tags. All experiments were conducted in accordance with the relevant laws and the guidelines provided by the Experimental Animal Center of South China Agricultural University. Furthermore, the experimental protocol for this study received review and approval from the Animal Experimentation Ethics Committee of South China Agricultural University (No. 2023b132).

Mice were fasted for 4 h but were provided free access to water. One hundred grams of QH were dissolved in 100 mL of distilled water, and established multi-dose group consisting of 3 to 10 times. The mice were divided into multiple groups, with four males and four females in each group, and were administered by oral gavages once a day at a dose of 20 mL/kg body weight. The observation of the overall condition, toxic symptoms, and mortality in mice was conducted for a period of seven days following treatment. Eventually, both the maximum and minimum tolerated doses were confirmed.

Based on the above experimental results, 4–6 dose groups were set between the maximum and minimum tolerated dose, with six males and six females in each group. The method was similar as above. Following the administration of QH, the mice were closely monitored for 24 h, with particular focus on the first 4 h, and were subsequently observed once daily for 7 consecutive days. During the entire experiment period, the weighed, mortality, behavioral pattern, appearance change, and illness signs of mice were observed and conducted once daily. At the end of the experiment, all mice that died from poisoning, as well as those that perished during natural death or were subjected to scheduled euthanasia using ether, underwent a complete necropsy. Institute of Cancer Research (ICR) mice received the same treatment procedures as mice.

### 2.6 Data analysis

Excel 2019, IBM SPSS Statistics 23, and SIMCA 14.1 were used for one-way ANOVA, correlation analysis, and principal component analysis (PCA) on the experimental data. GraphPad Prism 8.0.2 was used for heatmap plotting.

## 3 Results and discussion

### 3.1 Sensory evaluation of QH during aging time

**Fig. 1:**
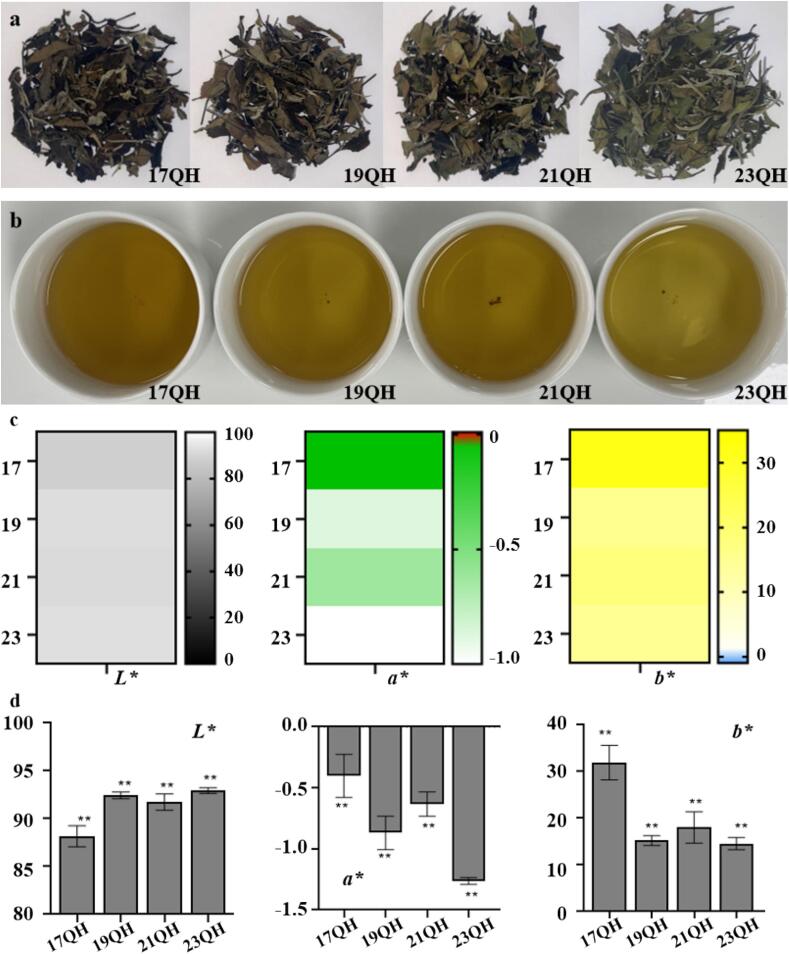
The appearance (a), liquor color (b), chromaticity values (c), and the correlation between chromaticity values and aging times (d) of QH. “* *” indicates a highly significant correlation (*p* < 0.01) between chromaticity values and aging times.

The sensory evaluation was conducted to assess the overall sensory characteristics of QH during aging time (Table S2). For appearance, there was certain regularity variation, with the most prominent difference observed in color. In the early stage of aging, the leaf surfaces transitioned from pale green to dark brown, while the underside shifted from emerald green to reddish-brown (Fig. 1a). Tea trichomes changed from white to yellowish-white after two years of storage. These changes were attributed to chlorophyll oxidation and lutein stability (Fan et al., 2021; Qiu et al., 2022). Consequently, the color of appearance appeared yellow or brown.

The color evolution of QH tea liquor during aging was quantified using the CIELAB color space. Chromaticity values showed significant variations (*p* < 0.05), with L* (brightness) decreasing from 93.04 to 87.05, while a* (redness) increased from −1.28 to −0.24 and b* (yellowness) rose from 13.54 to 35.31 (Fig. 1c, d). This resulted in a visible transition from pale brown to deep brown, aligning with Fan et al. (2021). These changes were primarily driven by water-soluble pigments, including TFs and thearubigins (TRs), as reported by Qiu et al. (2022). During the aging process, most theasinensins and proanthocyanidins showed a downward trend, whereas the more highly polymerized TFs and TBs increased (Zhao, Chen, et al., 2022). This observation suggested that aging might enhance the polymerization of polyphenols (Zhao, Zhang, et al., 2022), increase the amount of water-soluble pigments in the tea liquor, and contribute to the perceived quality and viscosity of tea.

For taste, QH in the early stage of aging was sweet and more thick, fresh and refreshing. During the aging process, the taste gradually transitioned to sweet and mellow, with a sweet and refreshing aftertaste. The bitterness decreased, allowing the taste to become increasingly sweet and mellow. As the aging time increased, the taste of QH turned into sweeter, mellower, and more harmonious, which was similar to the findings of Huang et al. (2023).

For aroma, QH in the early stage of aging was fragrant with dried fruit aroma and showed a subtle fragrance. During the aging process, the aroma gradually transformed into fragrances of floral and fruity, accompanied by hints of honey and dried fruit. The fresh aroma decreased; however, it transformed into woody and aged fragrances. The taste of tea was determined by the types and contents of non-volatile compounds in the dry tea (Huang et al., 2024). As the aging time increased, the taste of QH became sweeter and more harmonious, aligning with findings reported by Huang et al. (2023).

### 3.2 Quantitative analysis of the major non-volatile metabolites of QH during aging time

**Fig. 2:**
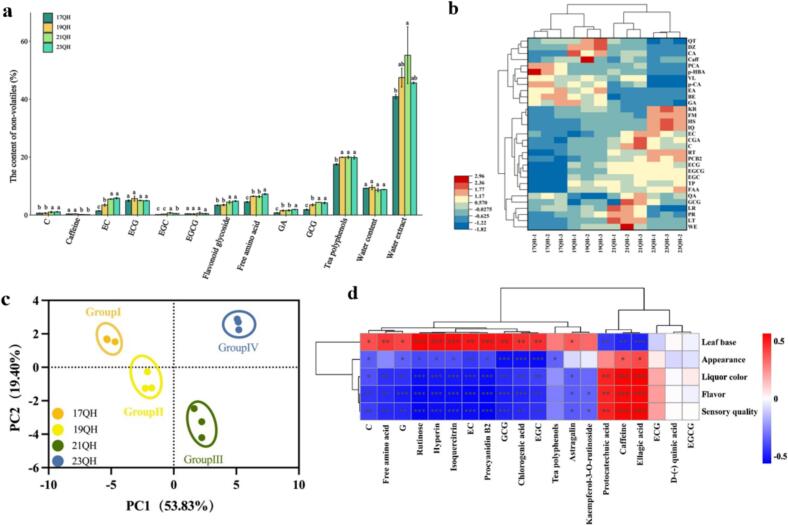
Quantitative analysis of the major non-volatile metabolites of QH during aging time. (a) the contents of non-volatile components in different storage years of Yunnan large white tea, (b) cluster analysis of non-volatile components, (c) principal component analysis, (d) the correlation analysis of the major non-volatiles with sensory quality. WC, water content; WE, water extract; FAA, free amino acids; TP, tea polyphenols; Caff, caffeine; QA, quinic acid; CA, caffeic acid; p-CA, p-coumaric acid; VX, vitexin; KF, kaempferol; p-HBA, p-hydroxybenzoic acid; ET, esculetin; DZ, daidzein; IQ, isoquercitrin; QT, quercetin; VR, vitexin-2-*O*-rhamnoside; AG, astragaloside; TA, tannic acid, PCA, protocatechuic acid; CGA, chlorogenic acid; PCB2, proanthocyanidin B2; RT, rutin; VL, vanillin; HS, hyperoside; KR, kaempferol-3-*O*-glucoside.

Given the observed discrepancy in taste, the samples were analyzed to quantify the concentrations of 42 key non-volatiles, including water extract, water content, tea polyphenols, free amino acids, caffeine, 7 monomers of catechin (GA, EGC, C, EC, EGCG, GCG, and ECG) (Fig. S1), and 20 monomers of flavonoid glycosides (*D*-(−) quinic acid, caffeic acid, *cis*-4-coumaric acid, vitexin, taxifolin, hydroxybenzoic acid, 6,7-dihydroxycoumarin, daidzein, isoquercitrin, astragalin, quercetin, vitexin 2-*O*-rhamnoside, tannic acid, original catechins, chlorogenic acid, procyanidin B2, rutin, vanillin, hypericin, and kaempferol-3-rutinoside) (Fig. S2, Table S3). Among them, the contents of various components in water extract, free amino acids, flavonoid glycosides, tea polyphenols, EGC, C, EC, EGCG, and ECG exhibited a gradual decline over the aging period. In contrast, the levels of GA increased gradually with the aging time, ranging from 0.15 % to 0.39 %. However, no significant trends were observed in water content, caffeine, and GCG (Fig. 2a). In addition, the clustering heatmap illustrated that 23QH & 21QH and 19QH & 17QH were respectively clustered together, indicating that their characteristics were similar (Fig. 2b).

A Pearson correlation analysis was performed to assess the relationship between aging time and the concentrations of 42 primary non-volatile compounds. The results showed that caffeine, GA, caffeic acid, p-coumaric acid, p-hydroxybenzoic acid, quercetin, vitexin 2-*O*-rhamnoside, tannic acid, protocatechuic acid, and vanillin were negatively correlated with the aging time. Whereas, the contents of amino acids, tea polyphenols, EGC, C, EC, EGCG, GCG, quinic acid, vitexin, kaempferol, isoquercitrin, astragaloside, chlorogenic acid, anthocyanin, rutin, hyperoside, and kaempferol-3-*O*-glucoside exhibited a positive correlation with aging time (Table S4). In conclusion, the majority of various metabolites were associated with flavonol and flavone glycosides (Wang, Liang, et al., 2022). The observed trends were consistent with those reported in previous studies assessing the impact of different aging durations on white tea (Wang, Wang, et al., 2022; Wang et al., 2024). With the exception of GA, caffeine, and GCG, the concentrations of major non-volatile metabolites decreased to varying degrees during the aging process. Gallic acid was likely produced from the degradation of gallic acid-catechins, such as EGCG and ECG. During the first year of storage, gallic acid remained relatively stable, followed by a sharp increase in the third year, aligning with findings from studies on aging durations (Fan et al., 2021). Caffeine exhibited minimal variation during aging or processing (Fan et al., 2021), whereas the concentrations of EGC and EGCG diminished to varying extents (Zhang et al., 2011), consistent with the results of this study.

Moreover, the major non-volatile metabolites were further examined by PCA, with the score plot illustrating the differences between the two groups (Fig. 2c). In the score chart (Table S5), the first and second principal components (PC1 and PC2) accounted for over 73 % of the total variance in the overall model, with the top five contributors in PC1 being rutin, anthocyanin B2, EGCG, coumaric acid, and GA (53.83 %). While, the scores of top five contributors were taxifolin, vitexin, vitexin-2-*O*-rhamnoside, water extract, and kaempferol-3-*O*-glucoside in PC2 (19.4 %). Collectively, the contents of the major non-volatile metabolites in QH during aging time were certain differences, with the most significant variations observed in rutin, anthocyanin B2, EGCG, coumaric acid, GA, quercetin, vitexin, vitexin-2-*O*-glucoside, and water extract (Zhao, Zhang, et al., 2022).

Subsequently, a Pearson correlation analysis was performed between sensory quality and the contents of the major non-volatiles to explore the associations (Fig. 2d). It could be observed that protocatechuic acid, caffeine, and ellagic acid were positively correlated to appearance liquor color and flavor of sensory quality, with caffeine and ellagic acid exhibiting the strongest correlation with liquor color and flavor (*r* > 0.87). While the three non-volatiles mentioned above were negatively correlated to leaf base, with ellagic acid having the extremely significant correlation (*r* = −0.83). Additionally, 12 non-volatiles were positively correlated to leaf base, with rutinose, procyanidin B2, hyperin, and isoquercitrin exhibiting the strongest correlation (*r* = 0.83). In addition to the four substances mentioned above, EC, EGC, and GCG demonstrated a significantly negative correlation with flavor, appearance, and liquor color (r > 0.87). However, tea polyphenols, ECG, EGCG, and *D*-(−) quinic acid didn't show any significant correlation with sensory quality. In conclusion, caffeine and ellagic acid primarily affected the liquor color and flavor of QH during aging time, while rutinose, procyanidin B2, hyperin, and isoquercitrin primarily influenced the leaf base of QH during aging time.

### 3.3 Analysis of volatile metabolites

#### 3.3.1 Electronic nose detection of QH during the aging time

**Fig. 3:**
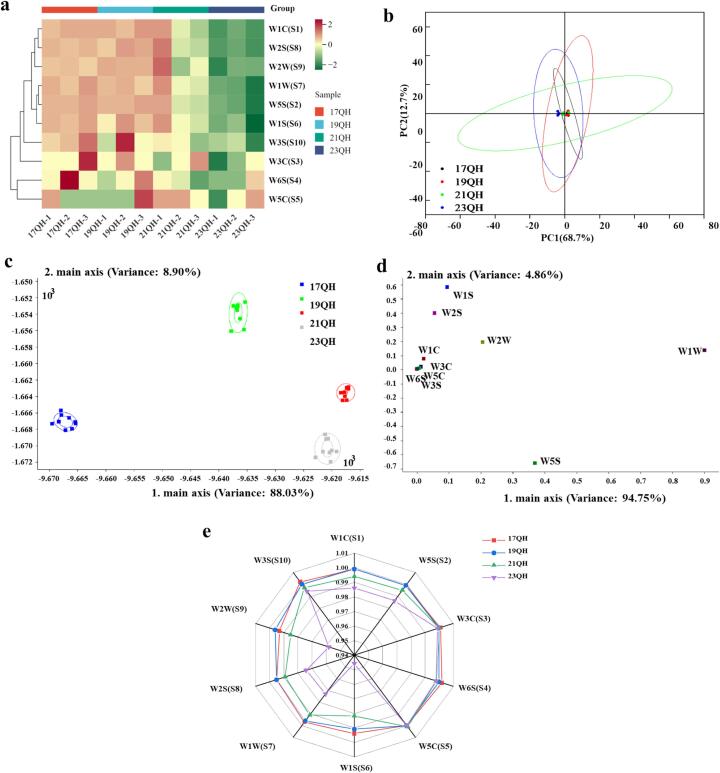
Stable response values of electronic nose sensors for samples of QH during aging time. (a) Heat map of electronic nose response values for samples of QH. (b) PCA score plot. (c) The LDA analysis score plot. (d) The loading analysis score plot. (e) Radar chart. Significant results based on Kruskal–Wallis test; “*” indicates *p* < 0.05; “**” indicates *p* < 0.01, and “NA” indicates *p* > 0.05. Expression: the size of the response values of different samples to the same sensor.

*E*-nose technology, a sensor system that simulates the human nose, has emerged as a promising and objective approach for the rapid identification of differences in tea aromas and the assessment of tea quality (Wen et al., 2023; Wijaya et al., 2024). In the current study, the results obtained from E-nose analysis indicated significant differences among the QH samples throughout the aging period (*p* < 0.05) (Fig. 3). The heatmap showed that all samples were divided into two major categories by cluster analysis, which is consistent with the classification of leaves by aging time (Fig. 3a). The W3S and W6S sensors demonstrated positive response values for the appropriate tea samples (17QH and 19QH) and negative response values for the unsuitable tea samples (21QH and 23QH). Unlike W1C, W1W, W1S, W2S, W2W, and W5S sensors showed intergroup differences in their response values to the four different samples.

Moreover, the normalized data obtained through z-score normalization from *E*-nose sensor response values were further analyzed by PCA. The sum contribution rates of PC1 (68.7 %) and PC2 (12.7 %) to the overall model exceeded 81 % in the score chart (Fig. 3b). The cumulative contribution rates of the principal components to the overall model exceeded 96 % in the LDA analysis chart (Fig. 3c). Additionally, the scores of QH at different aging times differed significantly with respect to PC1 (*p* < 0.05), which accounted for 88.03 % of the variance along the distribution (x-axis). Both 19QH and 21QH teas exhibited closer proximity to 23QH tea, while displaying greater distance from 17QH tea. The results indicated significant differences in the response values of various sensors within QH, demonstrating that *E*-nose could effectively distinguish the aroma of Yunnan large-leaf white tea during the aging process. The proximity of the sensor's response value to the coordinate origin on the loading graph indicates a higher representativeness of the principal component for that variable (Du et al., 2022).

The loading diagram demonstrated that W1S, W2S, and W1W sensors were located farthest from the x-axis origin, while W1W and W5S were positioned farthest from the y-axis (Fig. 3d). This indicated that the first group of sensors contributed significantly to the first principal component, whereas the latter two sensors contributed predominantly to the second principal component. These results suggested that sensors W1S, W2S, W1W, W5S played a significant role in Yunnan large-leaf white tea during aging process. Additionally, aromatic compounds, organic amines and hydrocarbon detected in the *E*-nose assay may serve as significant indicators for tea aroma. The radar chart revealed that the response values of sensors W1S/S6, W1W/S7, W2S/S8, and W2W/S9 significantly increased compared to those of 23QH tea (*p* < 0.05), whereas the response values of sensors W1C/S1, W5S/S2, and W3S/S10 showed a slight increase (Fig. 3e). Besides, the response values of sensor W3C/S3, W6S/S4, and W5C/S5 remained unchanged. An analysis of the compounds detected by these sensors revealed a significant increase in response values for alcohols, aldehydes, ketones, aromatic compounds, organic sulfides, sulfides, terpenes, and methyl compounds during the aging process.

The response signals of *E*-nose provide valuable insights into the profile of tea aroma compounds. Previous studies have demonstrated that the *E*-nose response for dark tea primarily consisted of aromatic compounds, organic amines, and hydrocarbons (Wen et al., 2023). Distinguishing factors for different grades of oolong tea included sulfides, hydrocarbons, methane, and alkanes (He et al., 2022), while nitrogen oxides and aromatic compounds were distinguishing factors for green tea (Yuan et al., 2019). Additionally, alcohols, aldehydes, ketones, aromatics, and organic sulfides were significant components in black tea (Xia et al., 2024). Therefore, the detection of aromatic compounds, organic amines, hydrocarbon in the *E*-nose assay may save as significant indicators for tea aroma, which was consistent with our findings.

In summary, these findings indicated that aging enhanced the levels of aromatic compounds (e.g., floral, fruity, and honey notes) and pungent (e.g., woody scents) in white tea. The trends of the response values of pungent compounds detected by the E-nose assay were consistent with the results of sensory evaluation. In this study, E-nose was able to significantly distinguish Yunnan large-leaf white tea aged for different durations, with all data demonstrating statistical significance *(p* < 0.05). Not all sensors significantly contributed to this process; therefore, we excluded W3C/S3, W6S/S4, and W5C/S5 from the subsequent analysis and concentrated on W1S/S6, W1W/S7, W2S/S8, and W2W/S9 as the primary sensors. Furthermore, the data obtained from E-nose data were consistent with the results of sensory evaluation, demonstrating not only the accuracy of E-nose detection but also the strong correlation between E-nose data and human sensory perception.

#### 3.3.2 Comparison of total volatile components in QH during aging

**Fig. 4:**
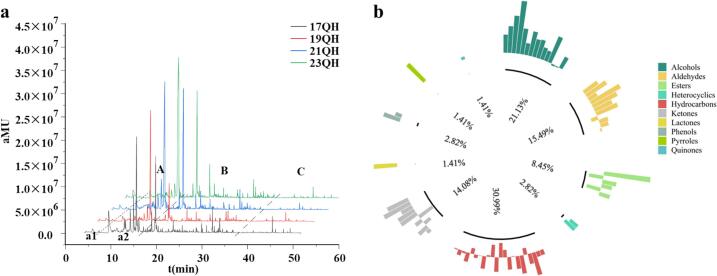
Comparison analysis of total volatile components in QH during aging time. (a) Total ion chromatogram. (b) Proportion of different classes of volatile compounds.

The analysis of volatile fingerprint of QH during the aging process revealed significant differences in the profiles of volatiles among the various piled white tea samples (Fig. 4a). As the aging years increased, the substances content of part a1 (e.g., n-hexanal) in low boiling point substance A increased, while the content of linalool in part a2 decreased and the content of linalool oxide increased. A total of 71 volatile compounds were detected in QH during aging periods, which were categorized according to their functional groups into alcohols, aldehydes, esters, hydrocarbons, heterocyclics, ketones, phenols, pyrroles, quinones, and lactones (Table 1). Among these, hydrocarbons were the most abundant aromatic compounds, accounting for 30.9 % of the total volatiles, followed by alcohols, aldehydes, ketones, and esters with proportions of 21.1 %, 15.5 %, 14.1 %, and 8.5 %, respectively (Fig. 4b). Additionally, two heterocyclics, two phenolics, pyrrole, quinone, and lactone were also identified with proportions of 9.9 % of the total volatiles. The quantities and proportions of volatiles in QH exhibited slight variations over the storage period (Table 1). Two prominent changes were observed during the storage of white teas: Several volatile compounds such as 1,7,7-trimethyl-bicyclo[2.2.1]hept-2-ene, hexanal, *endo*-borneol, (*E*)-1-(2,6,6-trimethyl-1,3-cyclohexadien-1-yl)-2-buten-1-one, linalool oxide III, and dihydroactinidiolide emerged in the aged white tea, whereas the peculiar substances including (*E*)-2-hexenal, *β*-myrcene, 2,6-dimethyl-2,4,6-octatriene, 2-methyl-decane, and 3-methyl-tridecane were no longer detected in new white tea. Most notably, linalool oxide II and methyl salicylate also existed in QH, while both of which were identified as unique compounds in new Fujian white tea (Ma et al., 2023). This observation may constitute a significant difference between Fujian white tea and Yunnan white tea.

**Table 1:** The content of volatiles in Yunnan large-leaf white tea during different storage years (μg/kg, on dry weight).

No	Volatiles	CAS	RT^a^	RI^b^	RI^c^	QI^d^^e^	17QH	19QH	21QH	23QH
	***Alcohols***									
1	Benzyl alcohol	000100–51-6	12.93	1038	1035	96	16.77 ± 8.35	N.D.	7.66 ± 0.44	7.05 ± 1.58
2	Linalool oxide I	005989–33-3	14.57	1071	1072	91	N.D.	15.92 ± 2.25	45.54 ± 2.1	24.02 ± 3.39
3	Linalool oxide II	034995–77-2	15.29	1088	1088	87	128.19 ± 4.1	50.63 ± 1.06	91.97 ± 2.96	22.37 ± 3.16
4	Linalool	000078–70-6	15.85	1102	1104	97	254.62 ± 18.35	389.74 ± 6.7	527.82 ± 11.59	676.31 ± 9.87
5	Phenylethyl alcohol	000060–12-8	16.50	1115	1114	94	49.4 ± 2.17	N.D.	13.72 ± 1.94	16.31 ± 1.64
6	*endo*-Borneol	000507–70-0	18.76	1164	1165	97	4.73 ± 0.67	4.86 ± 0.15	6.26 ± 0.18	N.D.
7	Linalool oxide III	014009–71-3	18.98	1169	1168	80	29.58 ± 2.05	11.23 ± 1.59	27.62 ± 3.91	N.D.
8	Linalool oxide IV	039028–58-5	19.23	1174	1183	91	N.D.	N.D.	16.36 ± 2.31	14.24 ± 2.01
9	Terpinen-4-ol	000562–74-3	19.30	1176	1175	96	34.95 ± 1.55	N.D.	N.D.	N.D.
10	*α*-Terpineol	000098–55-5	19.95	1190	1190	94	N.D.	16.06 ± 2.28	N.D.	N.D.
11	(*Z*)-3,7-dimethyl-2,6-Octadien-1-ol	000106–25-2	21.72	1229	1228	83	1.33 ± 0.19	N.D.	6.24 ± 1.74	5.78 ± 0.37
12	Geraniol	000106–24-1	22.92	1256	1255	94	8.21 ± 0.69	8.52 ± 0.75	28.32 ± 8.99	49.37 ± 6.72
13	1-Dodecanol	000112–53-8	32.26	1475	1476	91	N.D.	N.D.	1.79 ± 0.49	1.62 ± 0.48
14	2-Ethyl-2-methyl-tridecanol	1,000,115–66-1	32.87	1490	1490	90	N.D.	N.D.	3.39 ± 0.23	N.D.
15	Nerolidol	000142–50-7	35.76	1564	1566	93	5.73 ± 0.98	2.06 ± 0.11	4.25 ± 0.21	4.89 ± 1.05
	***Aldehydes***									
16	Hexanal	000066–25-1	4.65	803	802	97	9.52 ± 0.53	12.6 ± 1.05	6.48 ± 0.92	N.D.
17	(*E*)-2-hexenal	006728–26-3	5.99	852	850	98	N.D.	11.93 ± 1.68	37.59 ± 3.82	14.1 ± 9.2
18	2-Hexenal	000505–57-7	6.47	866	854	93	N.D.	N.D.	N.D.	12.14 ± 1.72
19	Benzaldehyde	000100–52-7	9.78	961	959	96	42.09 ± 5.96	42.66 ± 2.78	65.89 ± 1.27	42.99 ± 2.6
20	Benzeneacetaldehyde	000122–78-1	13.26	1043	1043	94	53.28 ± 1.67	8.11 ± 0.7	13.15 ± 1.86	15.1 ± 1.23
21	2,6,6-Trimethyl-1,3-cyclohexadiene-1-carboxaldehyde	000116–26-7	20.33	1198	1197	98	8.97 ± 1.27	7.99 ± 0.06	11.17 ± 0.41	8.6 ± 0.47
22	Decanal	000112–31-2	20.69	1206	1206	99	18.88 ± 2.88	13.42 ± 3.95	12.04 ± 0.25	14.56 ± 3.18
23	3,4-Dimethyl-benzaldehyde	005973–71-7	21.04	1214	1214	93	N.D.	1.82 ± 0.26	N.D.	N.D.
24	*β*-Cyclocitral	000432–25-7	21.27	1219	1220	98	N.D.	1.56 ± 0.22	6.15 ± 0.51	3.73 ± 0.13
25	*α*-Ethylidene-benzeneacetaldehyde	004411–89-6	23.67	1272	1276	98	3 ± 0.42	N.D.	6.88 ± 0.98	N.D.
26	2-Butyl-2-octenal	013019–16-4	28.06	1374	1372	89	N.D.	N.D.	1.17 ± 0.17	2.1 ± 0.29
	***Esters***									
27	Methyl salicylate	000119–36-8	20.10	1193	1193	97	143.43 ± 14.39	92.04 ± 2.51	390.23 ± 7.89	255.3 ± 23.94
28	Dodecyl acrylate	002156–97-0	41.03	1675	1675	91	N.D.	N.D.	0.74 ± 0.10	N.D.
29	1,2-Benzenedicarboxylic acid, bis(2-methylpropyl) ester	000084–69-5	46.41	1867	1868	91	6.98 ± 2.41	4.59 ± 1.00	5.23 ± 0.30	3.5 ± 0.14
30	Hexadecanoic acid, methyl ester	000112–39-0	48.11	1932	1927	99	5.85 ± 0.34	2.32 ± 0.04	2.85 ± 0.68	2.71 ± 0.46
31	Dibutyl phthalate	000084–74-2	48.90	1967	1967	97	N.D.	N.D.	0.44 ± 0.06	0.31 ± 0.04
32	Hexadecanoic acid, ethyl ester	000628–97-7	49.53	1996	1997	99	6.65 ± 0.84	1.73 ± 0.39	2.61 ± 0.15	2.98 ± 0.53
	***Hydrocarbons***									
33	*β*-Myrcene	000123–35-3	11.03	991	992	95	N.D.	N.D.	N.D.	7.31 ± 1.03
34	p-Cymene	000099–87-6	12.39	1023	1024	97	N.D.	2.07 ± 0.29	5.16 ± 0.87	N.D.
35	d-limonene	005989–27-5	12.56	1027	1026	99	6.63 ± 0.27	15.99 ± 1.71	22.08 ± 8.52	11.25 ± 1.47
36	3,6-Dimethyl-decane	017312–53-7	13.91	1057	1057	90	N.D.	N.D.	9.26 ± 0.49	3.35 ± 0.47
37	2,6-Dimethyl-2,4,6-octatriene	000673–84-7	17.19	1129	1128	95	N.D.	N.D.	N.D.	1.8 ± 0.18
38	Dodecane	000112–40-3	20.42	1200	1200	96	10.83 ± 3.67	5.84 ± 0.27	N.D.	15.52 ± 0.85
39	1,7,7-Trimethyl-bicyclo[2.2.1]hept-2-ene	000464–17-5	21.65	1227	1227	96	10.65 ± 0.31	1.92 ± 0.12	5.28 ± 0.71	N.D.
40	1,3-Bis(1,1-dimethylethyl)-benzene	001014–60-4	22.81	1253	1253	95	N.D.	0.65 ± 0.09	1.72 ± 0.55	1.13 ± 0.38
41	2-methyl-Dodecane	001560–97-0	23.28	1264	1263	83	N.D.	N.D.	N.D.	16.99 ± 2.40
42	1,2-dihydro-1,1,6-trimethyl-Naphthalene	030364–38-6	27.06	1350	1349	95	1.63 ± 0.23	N.D.	N.D.	N.D.
43	3-Methyl-tridecane	006418–41-3	27.93	1370	1371	95	N.D.	N.D.	N.D.	0.73 ± 0.10
44	Tetradecane	000629–59-4	29.18	1399	1400	97	6.74 ± 0.33	2.29 ± 0.32	5.71 ± 1.07	5.95 ± 0.32
45	Pentadecane	000629–62-9	33.26	1499	1500	96	N.D.	0.5 ± 0.07	N.D.	0.47 ± 0.07
46	2-Methyl-pentadecane	001560–93-6	36.24	1536	1535	86	N.D.	N.D.	N.D.	3.23 ± 0.35
47	Hexadecane	000544–76-3	37.14	1599	1600	98	4.33 ± 0.33	3.14 ± 0.16	4.17 ± 0.59	4.78 ± 1.77
48	2-methyl-Hexadecane	001560–92-5	39.82	1665	1665	89	2.42 ± 0.43	N.D.	1.84 ± 0.78	N.D.
49	Heptadecane	000629–78-7	41.22	1495	1700	96	0.39 ± 0.05	N.D.	2.64 ± 0.64	2.67 ± 0.38
50	1-Heptadecene	006765–39-5	41.31	1702	1692	95	N.D.	N.D.	1.38 ± 0.20	0.71 ± 0.10
51	8-hexyl-Pentadecane	013475–75-7	42.99	1756	1755	80	N.D.	11.02 ± 1.56	N.D.	N.D.
52	3-Methyl-heptadecane	006418–44-6	43.56	1773	1771	90	1.27 ± 0.54	0.44 ± 0.06	1.41 ± 0.58	1.75 ± 0.17
53	Octadecane	000593–45-3	44.39	1780	1800	93	2.06 ± 0.31	1.37 ± 0.19	1.41 ± 0.90	2.09 ± 0.70
54	2,6,10,14-Tetramethyl-hexadecane	000638–36-8	44.66	1808	1809	93	N.D.	N.D.	3.7 ± 0.52	0.58 ± 0.08
	***Heterocyclics***									
55	3,4-Dimethoxytoluene	000494–99-5	22.24	1230	1246	94	1.28 ± 0.35	N.D.	N.D.	N.D.
56	(4-acetylphenyl)phenylmethane	000782–92-3	40.62	1685	1687	83	0.81 ± 0.12	N.D.	N.D.	N.D.
	***Ketones***									
57	6-Methyl-5-hepten-2-one	000110–93-0	10.91	991	985	96	4.08 ± 0.58	N.D.	1.93 ± 0.27	N.D.
58	1-Ethyl-2,5-pyrrolidinedione	002314–78-5	17.51	1136	1137	90	8.4 ± 1.19	N.D.	3.47 ± 0.49	1.82 ± 0.91
59	5,5-Dimethyl-3-oxo-1-cyclohexene-1-carboxaldehyde	056621–35-3	17.80	1219	1220	90	1.6 ± 0.23	N.D.	N.D.	N.D.
60	(*E*)-1-(2,6,6-trimethyl-1,3-cyclohexadien-1-yl)-2-buten-1-one	023726–93-4	28.49	1384	1383	97	4.95 ± 0.69	1.38 ± 0.21	3.4 ± 0.52	N.D.
61	4-(2,6,6-Trimethyl-1-cyclohexen-1-yl)-2-butanone	017283–81-7	30.73	1437	1439	99	0.58 ± 0.08	N.D.	N.D.	N.D.
62	*α*-Ionone	000127–41-3	30.29	1427	1427	98	16.47 ± 4.14	5.31 ± 0.16	8.55 ± 0.56	5.53 ± 0.44
63	(*E*)-6,10-dimethyl-5,9-Undecadien-2-one	003796–70-1	31.37	1453	1454	95	13.74 ± 5.12	6.67 ± 0.08	8.39 ± 1.05	6.74 ± 1.33
64	4-(2,6,6-Trimethylcyclohexa-1,3-dienyl)but-3-en-2-one	001203–08-3	32.56	1482	1485	97	9.82 ± 1.16	4.24 ± 0.13	1.87 ± 0.25	0.47 ± 0.06
65	4-(2,6,6-trimethyl-1-cyclohexen-1-yl)-3-Buten-2-one	014901–07-6	32.67	1485	1488	98	44.84 ± 15.05	17.34 ± 0.1	31.59 ± 0.08	23.37 ± 4.02
66	6,10,14-Trimethyl-2-pentadecanone	000502–69-2	45.70	1843	1846	99	20.62 ± 1.28	12.17 ± 2.25	12.14 ± 3.49	11.32 ± 1.04
	***Phenols***									
67	Butylated hydroxytoluene	000128–37-0	33.76	1512	1512	98	4.35 ± 0.62	2.86 ± 0.41	12.63 ± 8.7	6.3 ± 0.05
68	2,4-Di-tert-butylphenol	000096–76-4	33.78	1513	1513	91	5.03 ± 0.13	N.D.	1.39 ± 0.19	1.86 ± 0.07
	***Pyrroles***									
69	1-Ethyl-1H-pyrrole-2-carboxaldehyde	002167–14-8	13.55	1048	1048	91	33.04 ± 4.67	N.D.	29.76 ± 1.41	N.D.
	***Quinones***									
70	2,6-Bis(1,1-dimethylethyl)-2,5-cyclohexadiene-1,4-dione	000719–22-2	31.86	1465	1458	99	N.D.	N.D.	1.32 ± 0.26	1.46 ± 0.41
	***Lactones***									
71	(*R*)-5,6,7,7a-tetrahydro-4,4,7a-trimethyl-2(4H)-Benzofuranone	017092–92-1	34.27	1525	1525	97	32.01 ± 9.95	10.71 ± 0.40	14.89 ± 0.77	N.D.

a RT: retention time.

b RT: retention index (RI) was calculated using n-alkanes (C8–C40) under the same chromatographic conditions applied to the detected volatile compounds.

c RT: data were obtained from the NIST database.

d QI: quantitation ion.

e N.D.: not found.

Variations in the total volatile contents of QH were observed during the aging process. As the aging time increased, the volatile compounds of QH displayed a decreasing trend, particularly among alcohols, esters, and hydrocarbons, whereas ketones demonstrated an increasing trend. The total volatiles in 23QH teas decreased from 1339.2 μg/kg to 1081.32 μg/kg after five years of aging. Alcohol was one of the most common volatile substances in QH teas, with the highest content in 23QH teas, accounting for over 61 % of the total volatiles, while it decreased by 35.09 % in 17QH teas. These findings were similar to the results of Wang, Wang, et al. (2022) who reported that the main volatile components of white tea were alcohols and esters, and the alcohols content were the highest during early aging time. Methyl salicylate, characterized by almond and fruity notes, has the highest content among volatile compounds, which decreased with extended storage duration. Following short-term storage, the concentration of volatile ketones in white tea increased (Wang, Wang, et al., 2022; Wang et al., 2024), aligning with the findings of this study. Notably, *β*-ionone, known for its woody aroma, exhibited the highest concentration among these compounds, which further increased during the aging process.

In contrast to alcohols, the concentration of aldehydes consistently increased during the first two years of aging, contributing significantly to the total volatiles in QH aging. However, aldehydes gradually decreased as the duration of aging extended. The increase in aldehydes during the initial storage period may be attributed to lipid oxidation (Wang, Li, et al., 2022). In addition, the content of phenylacetaldehyde with green leaf and hyacinth aroma along with benzaldehyde with the aroma of bitter almonds increased with aging years (Table 1). While 2-hexenal contributed to the fresh and tender aroma and was recognized as one of key components in white tea, it was only detected in the 23QH samples. Aldehydes, which are the most abundant volatiles (Chen et al., 2019), also increased in concentration during the storage of white tea (Wang et al., 2024). These results indicated that the primary volatile compounds, alcohols and aldehydes, undergo a transition in their aroma characteristics from grassy, light, and tender fragrances to woody, fruity, sweet, and medicinal aromas during the aging process of Yunnan large leaf white tea.

#### 3.3.3 Screening for key characteristics of volatile components in QH during aging

**Fig. 5:**
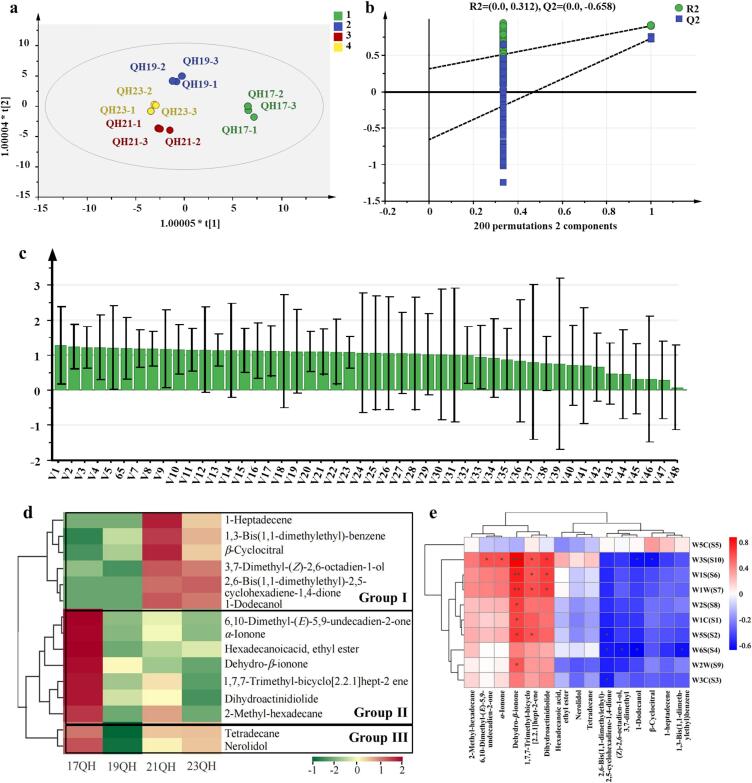
Screening for key characteristics of volatile components in QH during aging. (a) Scores plots of PLS-DA. (b) Cross-validation plot by 200 permutation tests. (c) Volatiles with VIP > 1 for QH during different aging times (detailed information was recorded in Table S4). (d) Clustering heat map. (e) Correlation analysis of the key volatiles with *E*-nose. “^⁎^”, “^⁎⁎^”, and “^⁎⁎⁎^” indicate the correlation coefficient with statistically significant value of *p* < 0.05, *p* < 0.01, and *p* < 0.001, respectively.

Partial least squares discriminant analysis (PLS-DA) was employed as a supervised recognition pattern aimed at maximizing the differentiation, identification, and classification among samples (Zhou et al., 2024). The PLS-DA model generated from this analysis demonstrated strong interpretive and predictive capabilities, with R^2^X, R^2^Y, and Q^2^ values all greater than 0.88 (Fig. 5a). Additionally, the validity and reliability of the PLS-DA model were confirmed through the permutation test (200 times), without overfitting (R^2^ = 0.312, Q^2^ = − 0.658), which suggested that the OPLS-DA model was statistically significant and relevant to the analysis of tea flavor in terms of identifying (Fig. 5b).

Variable importance in the Projection (VIP) was a measure of the weight of variables in the OPLS-DA model. Volatile substances with higher VIP values contributed more to the overall aroma of tea samples. A total of 48 volatile components were obtained based on the criteria with VIP > 1 (Fig. 5c, Table S4). To further analyze the contribution rate of different aroma compounds in distinguishing QH during the aging process, 15 characteristic aroma components were screened according to the criteria with VIP > 1 and *p* < 0.05. These volatiles included 3 ketones (*α*-ionone, geranylacetone, and dehydro-*β*-ionone), 1 aldehyde (*β*-cyclocitral), 2 esters (dihydroactinidiolide and hexadecanoic acid ethyl ester), 3 alcohols (nerol, lauryl alcohol, and nerolidol), 5 hydrocarbons, and 1 quinone (Table 2). Additionally, the hierarchical cluster analysis was performed to summarize the variations in differential volatiles during the aging of QH across various time points. These volatiles were classified into three groups, each exhibiting distinct trends (Fig. 5d). In group 1, volatile compounds showed a high level in 23QH teas, then peaked after 3-year storage (21QH tea), followed by a subsequent decline as storage duration extended (19QH and 17QH teas). In group 2, the concentration of volatiles increased after 3 years of storage, then decreased after storage for 5 years, and peaked after 7 years of storage. In group 3, volatile concentrations rapidly increased after storage for 3 years and maintained a higher level before swiftly declining, ultimately reaching their peak at 7 years of storage. These volatiles identified from QH exhibited distinct trends of variation during storage, suggesting their potential as biomarkers for distinction of white tea under different aging treatments. Furthermore, the variations in concentration during storage highlighted the responsible of crucial volatiles for the unique flavor of QH during the aging process.

**Table 2:** Relative content of volatile components based on OPLS-DA model (μg/kg, on dry weight).^a^, ^b^

NO	Volatiles	17QH	19QH	21QH	23QH	*p*-value	VIP
1	*β*-Cyclocitral	N.D.	1.56	6.15	3.73	0.02	1.22
2	1,7,7-Trimethyl-bicyclo[2.2.1]hept-2-ene	10.65	1.92	5.28	N.D.	N.D.	1.10
3	(*Z*)-2,6-octadien-1-ol, 3,7-dimethyl	1.33	N.D.	6.24	5.78	0.02	1.21
4	1,3-Bis(1,1-dimethylethyl)-benzene	N.D.	0.65	1.72	1.13	0.14	1.10
5	Tetradecane	6.74	2.29	5.71	5.95	0.37	1.05
6	*α*-Ionone	16.47	5.31	8.55	5.53	0.02	1.23
7	6,10-Dimethyl-(*E*)-5,9-undecadien-2-one	13.74	6.67	8.39	6.74	0.15	1.18
8	2,6-Bis(1,1-dimethylethyl)-2,5-cyclohexadiene-1,4-dione	N.D.	N.D.	1.32	1.46	0.01	1.13
9	1-Dodecanol	N.D.	N.D.	1.79	1.62	0.01	1.16
10	Dehydro-*β*-ionone	9.82	4.24	1.87	0.47	N.D.	1.19
11	Dihydroactinidiolide	32.01	10.71	14.89	N.D.	0.01	1.16
12	Nerolidol	5.73	2.06	4.25	4.89	0.03	1.02
13	2-Methyl-hexadecane	2.42	N.D.	1.84	N.D.	0.01	1.18
14	1-Heptadecene	N.D.	N.D.	1.38	0.71	0.14	1.12
15	Hexadecanoic acid, ethyl ester	6.65	1.73	2.61	2.98	0.02	1.09

a The relative content of volatile components with VIP > 1 and *p* < 0.05 was analyzed using the OPLS-DA model.

b N.D.: not found.

To investigate the impact of aging on volatiles, a Pearson correlation analysis was further performed to assess the relationship between *E*-nose and 15 key volatiles. As described in Fig. 5e, dehydro-*β*-ionone was positively correlated to W3S, W5S, W1C, W1W, W2S, W2W, and W1S, with the strongest correlation observed specifically with W3S (*r* = 0.84). Meanwhile, dihydroactinidiolide, *α*-ionone, 6,10-dimethyl-(*E*)-5,9-undecadien-2-one, and 1,7,7-trimethyl-bicyclo[2.2.1]hept-2-ene exhibited a significantly positively correlation with W3S (*r* > 0.6). These results indicated that E-nose was highly sensitive to alkane compounds, which contributed to the identification of key aromatic volatiles during the aging process of white tea.

In particular, trans-*β*-ionone (Wang, Wu, et al., 2020), characterized by its floral note and exceptionally low odor threshold of 0.007 ppb in water, is produced through the degradation of carotenoids catalyzed by carotenoid cleavage dioxygenases (Wang, Wu, et al., 2020; Wang, Zhang, et al., 2020). It is widely regarded as a critical odorant that plays a significant role in defining the aromatic profile of white tea, green tea, oolong tea, and black tea (Liu et al., 2022). In addition, dehydro-*β*-ionone, an oxide of trans-*β*-ionone, served as a key volatile compound during the aging process of QH, which contributed to the woody notes in the overall aroma. Furthermore, dihydroactinidiolide and *β*-cyclocitral significantly enhanced the floral fragrance of QH. These findings emphasized the essential role of carotenoids as fragrance precursors to the characteristic aroma of QH.

### 3.4 Aflatoxins determination and safety evaluation of QH

The degradation of AFs was monitored in QH teas across different storage periods. The results demonstrated that no AF-B and -G was detected in QH tea at any aging stage (Fig. S3).

To obtain comprehensive information regarding the quality components of QH, the concentrations of the measured compounds were presented in Table 3. Among these, the concentrations of the three main bioactive components, free amino acids, tea polyphenols, and caffeine, in 17QH teas decreased by 37.29 %, 11.69 %, and 0.99 %, respectively, compared to those in 23QH teas. In addition, the levels of water extracts, free amino acids, tea polyphenols, and caffeine in white tea after 7-year storage were higher than those in 1-year aged white tea, indicating that the selected tea samples have good representativeness, and these ingredients were the basis to their beneficial effects on health in QH.

**Table 3:** Quality components and LD_50_ of QH.^a^

Samples	WC/%	WE/%	FACs/%	TPs/%	Caf/%	LD_50_/k·kg^−1^	95 % confidence limit/k·kg^−1^	Caf of LD_50_ /mg·kg^−1^	TPs of LD_50_ /mg·kg^−1^
17QH	9.26 ± 0.04	40.88 ± 0.72	4.54 ± 0.17	17.53 ± 0.00	4.96 ± 0.10	17.17	14.82–19.92	851.49	301.06
23QH	8.82 ± 0.09	45.63 ± 0.31	7.24 ± 0.22	19.85 ± 0.02	5.01 ± 0.16	15.09	13.55–17.72	857.78	299.52

a WC: water content; WE: water extract; FACs: Free amino acids; Caf: caffeine; TP: tea polyphenol.

**Fig. 6:**
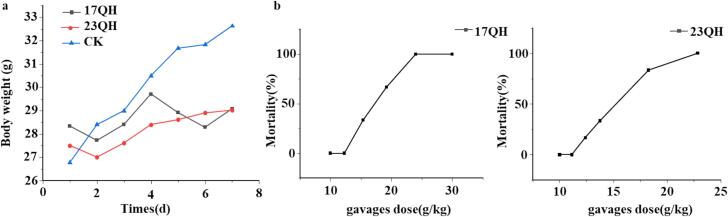
Effect of QH on body weight (a) and mortality (b) in the oral acute toxicity study (*n* = 6).

For safety evaluation, acute oral toxicity serves as an important index to identify the harmful effects of new resource foods. Maximum tolerated dose (MTD) showed that none of the mice died at minimum tolerated dose of 10.00 g/kg·bw within a period of 7 days, and their activity increased without any observable toxic reactions. In contrast, all mice succumbed at the maximum tolerated doses of 29.98 g/kg·bw for 17QH and 22.86 g/kg·bw for 23QH. Then, 6 doses were set between the maximum tolerated dose and minimum tolerated dose in an equal ratio of 0.8 for the acute toxicity test. LD_50_ values for 17QH and 23QH teas were 17.17 g/kg·bw and 15.09 g/kg·bw (Table 3), respectively, which indicated that LD_50_ was greater than 5000 mg/kg·bw both 1-year and 7-year aged white tea according to the toxicity grading standards, thereby confirming that Yunnan large-leaf white tea was indeed nontoxic. In addition, the body weight and mortality of all mice in both the control and treated groups (17QH and 23QH teas) were shown in Fig. 6, respectively. The body weight of surviving mice in the QH groups initially decreased but then rapidly increased after 4 days of gavage, in comparison to the control group. Although the concentration of white tea administered by oral gavages once a day was close to the LD_50_, it had a significant impact on the body weight of mice, and might cause toxicity due to excessive concentration of white tea, resulting bodily loss.

To further investigate the potential lethal causes associated with white tea, we analyzed the relationship between the LD_50_ and the concentrations of caffeine and tea polyphenols. The content of caffeine in two tea samples administered at LD_50_ via gavage was measured at 851.49 mg/kg·bw and 857.78 mg/kg·bw, respectively (Table 3), which were significantly higher than the LD_50_ of food-grade caffeine (127 mg/kg·bw). While two tea samples were administered with LD_50_ by gavage, the content of tea polyphenols was 301.06 mg/kg·bw and 299.52 mg/kg·bw, respectively, which was lower than the LD_50_ of food-grade tea polyphenols (3160 mg/kg·bw). It could be inferred that excessive concentration of caffeine can cause mouse death and tea polyphenol poisoning. The study reported by Wu et al. who found that the content of caffeine in Liubao tea has exceeded its LD_50_ (Wu et al., 2017). Furthermore, the mice exhibited symptoms of poisoning, including abnormal postures, decreased activity, difficulty in breathing, and sparse hair, with death occurring 1 to 2 h later compared to the control group. Necropsy revealed abnormal phenomena of gray red and slight whitening in the liver of some mice. The sites of poisoning may be associated with the central nervous and respiratory systems according to the animal response indicators of acute toxicity tests. Thes findings suggested that excessively high concentration of caffeine was likely to result in damage to the central nervous system (Huang et al., 2014).

## 4 Conclusion

In the current study, the aroma profiles, quality components, and safety assessment of Yunnan large-leaf white tea stored for different years were investigated using sensory evaluation, GC–MS, *E*-nose, acute toxicity in mice. The sensory evaluation indicated that storage duration significantly impacted the color, aroma, and taste of white tea. The contents of non-volatile components, such as free amino acids, EGC, and EGCG, demonstrated a downward trend as storage years increased, whereas those of p-hydroxybenzoic acid, aesculetin, and vanillin exhibited an upward trend. Additionally, 15 characteristic aroma components, including *α*-ionone, *β*-cyclocitral, tetradecane, dehydro-*β*-ionone, 1,7,7-trimethyl-bicyclo[2.2.1]hept-2-ene, 3,7-dimethyl-(*Z*)-2,6-octadien-1-ol, 1,3-bis(1,1-dimethylethyl)-benzene, 6,10-dimethyl-(*E*)-5,9-undecadien-2-one, dihydroactinidiolide, 2,6-bis(1,1-dimethylethyl)-2,5-cyclohexadiene-1,4-dione, 1-dodecanol, nerolidol, hexadecanoic acid, 2-methyl-hexadecane, 1-heptadecene, and ethyl ester, were identified as the crucial volatiles in Yunnan large-leaf white tea from different storage years. Furthermore, AF levels in QH teas remained undetected or below the threshold, irrespective of aging time; meanwhile, aged white teas for 1 and 7 years were classified as practically non-toxic, with LD_50_ values far exceeding the safe standard of 5000 mg/kg·bw. In conclusion, these findings provide a scientific foundation for the quality assessment and storage management of white tea.

## Ethics and consent

This study involves human or animal testing. Human sensory evaluation does not require ethical permission, and all panelists gave consent to participate in this research.

## CRediT authorship contribution statement

**Caibi Zhou:** Writing – review & editing, Writing – original draft, Validation, Software, Methodology, Investigation, Conceptualization. **Yongshi Chen:** Writing – original draft, Methodology, Conceptualization. **Mengling Chen:** Supervision, Resources, Formal analysis, Data curation. **Mei Xu:** Writing – review & editing, Supervision, Resources. **Dongwei Zhao:** Visualization, Software, Investigation, Conceptualization. **Long Huang:** Supervision, Investigation, Conceptualization. **Wenpin Chen:** Writing – review & editing, Supervision, Resources, Project administration, Funding acquisition.

## Funding

This research was supported by 10.13039/501100001809NSFC (31270725) and Enterprise Horizontal Project (Industrial development technology service for intelligent tea cabinet).

## Declaration of competing interest

There are none to declare.

## Data Availability

Data will be made available on request.

## Appendix A Supplementary data

Supplementary material 1 Image 1

Supplementary material 2 Image 2
