# Supplementary material for: Flavor changes of Yunnan large-leaf cultivar white tea during different aging periods
Source: Food Chem X. 2025 Jul 29;29:102846. doi: 10.1016/j.fochx.2025.102846 (PMC12357289; doi:10.1016/j.fochx.2025.102846)
Supplement: Supplementary file 1 — Supplementary material 1 [file mmc1.docx]

17QH-1

17QH-2

17QH-3

19QH-1

19QH-2

19QH-3

21QH-1

21QH-2

21QH-3

23QH-1

23QH-2

23QH-3

**Figure S1.** The chromatograms for flavonoid glycosides of QH teas in each sample.

17QH-1


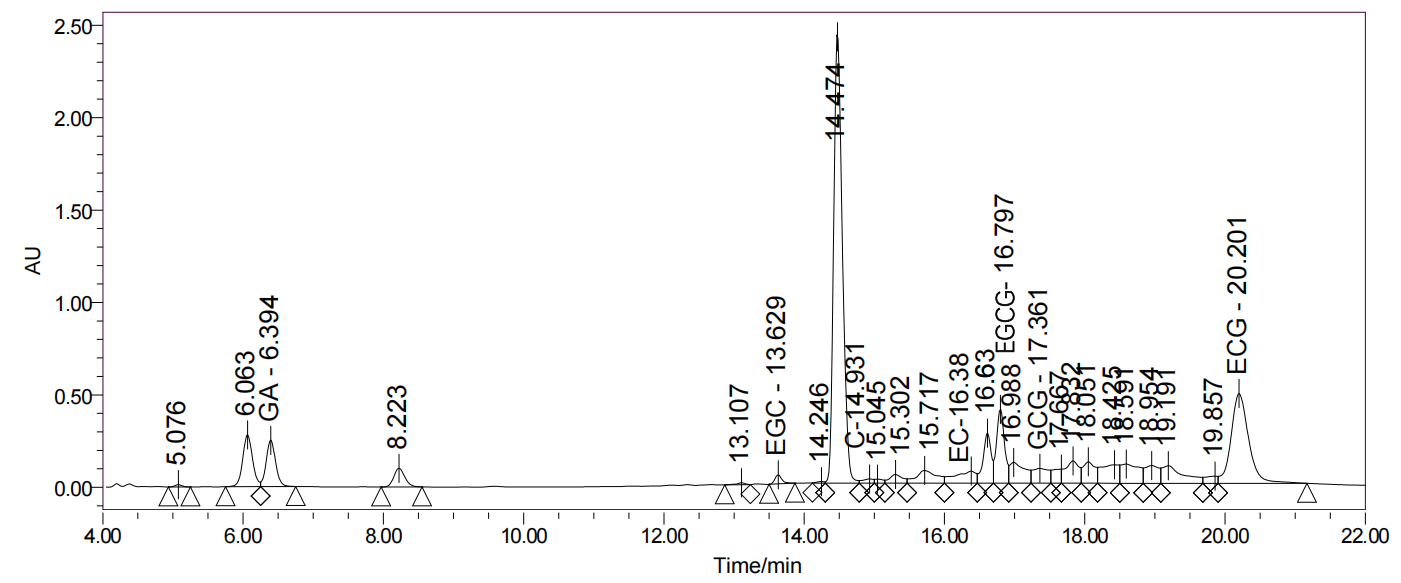


17QH-2


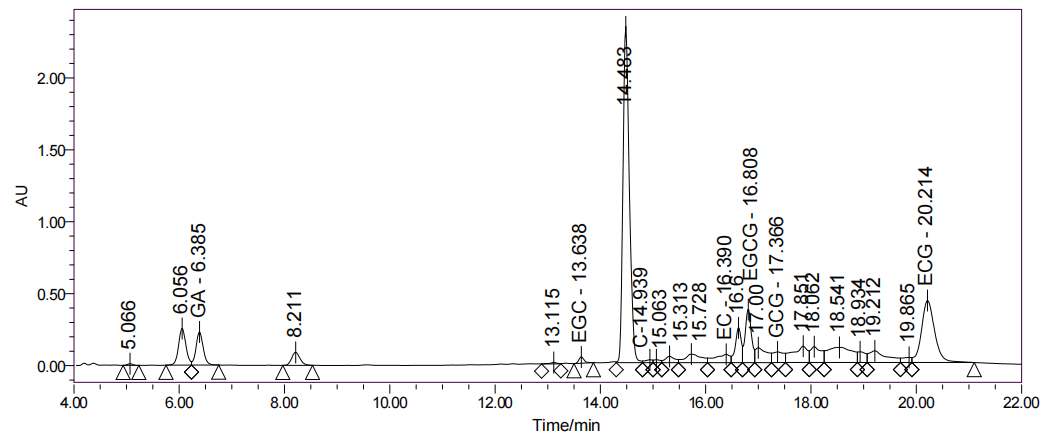


17QH-3


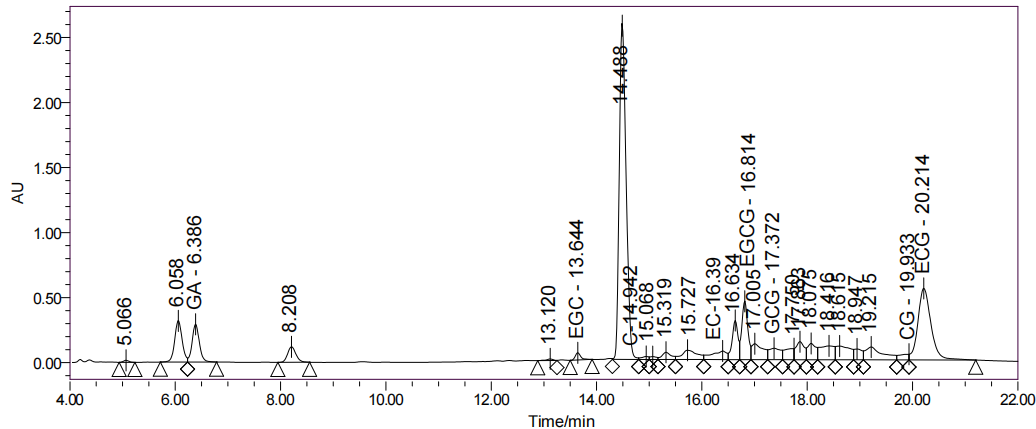


19QH-1


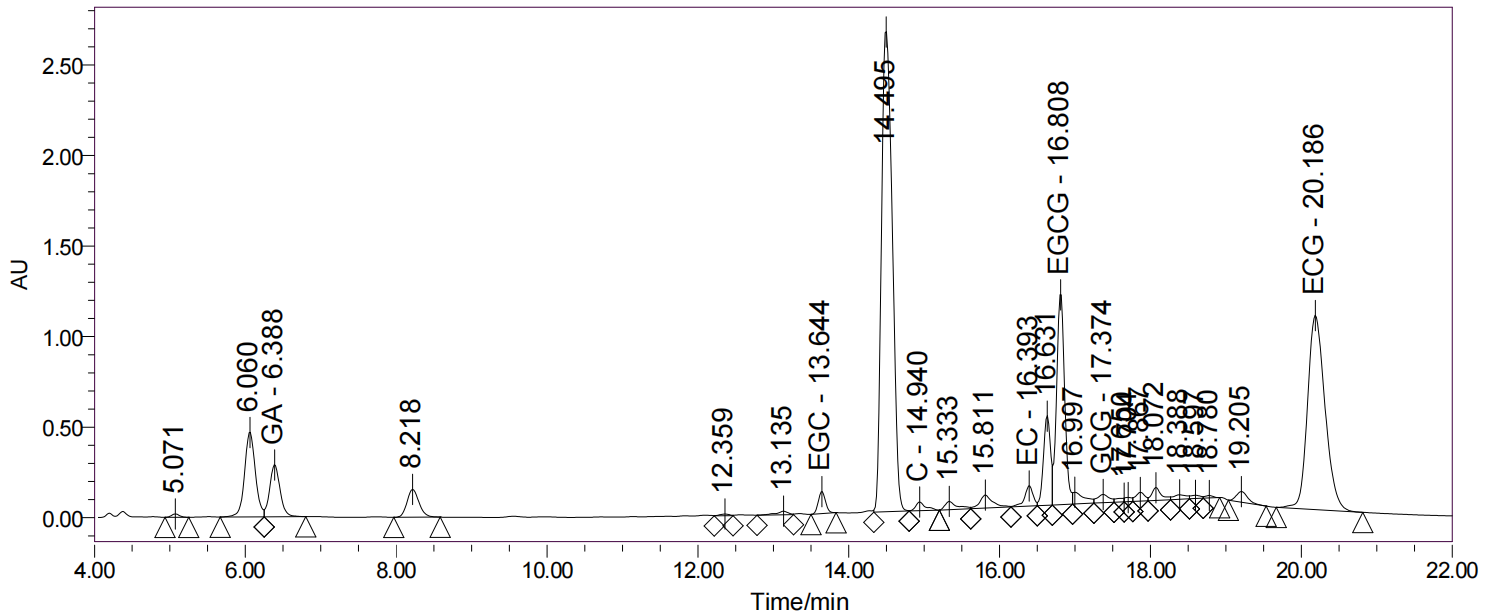


19QH-2


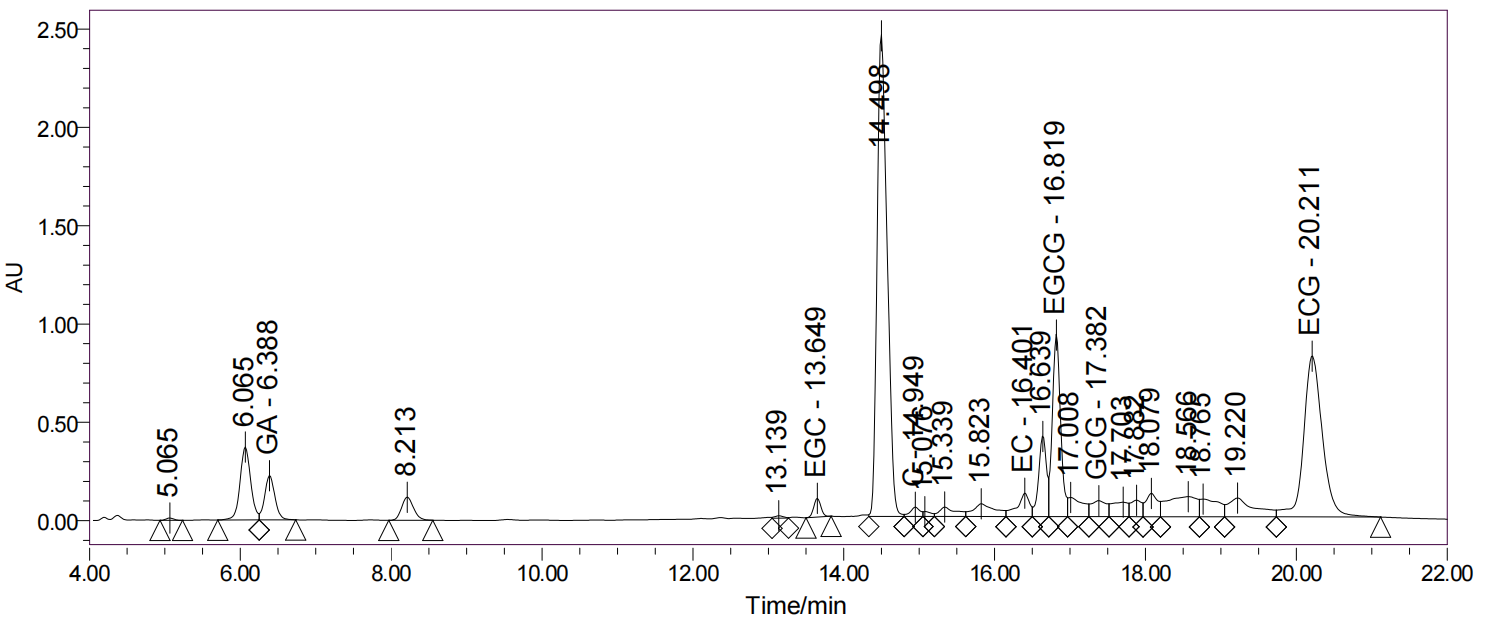


19QH-3


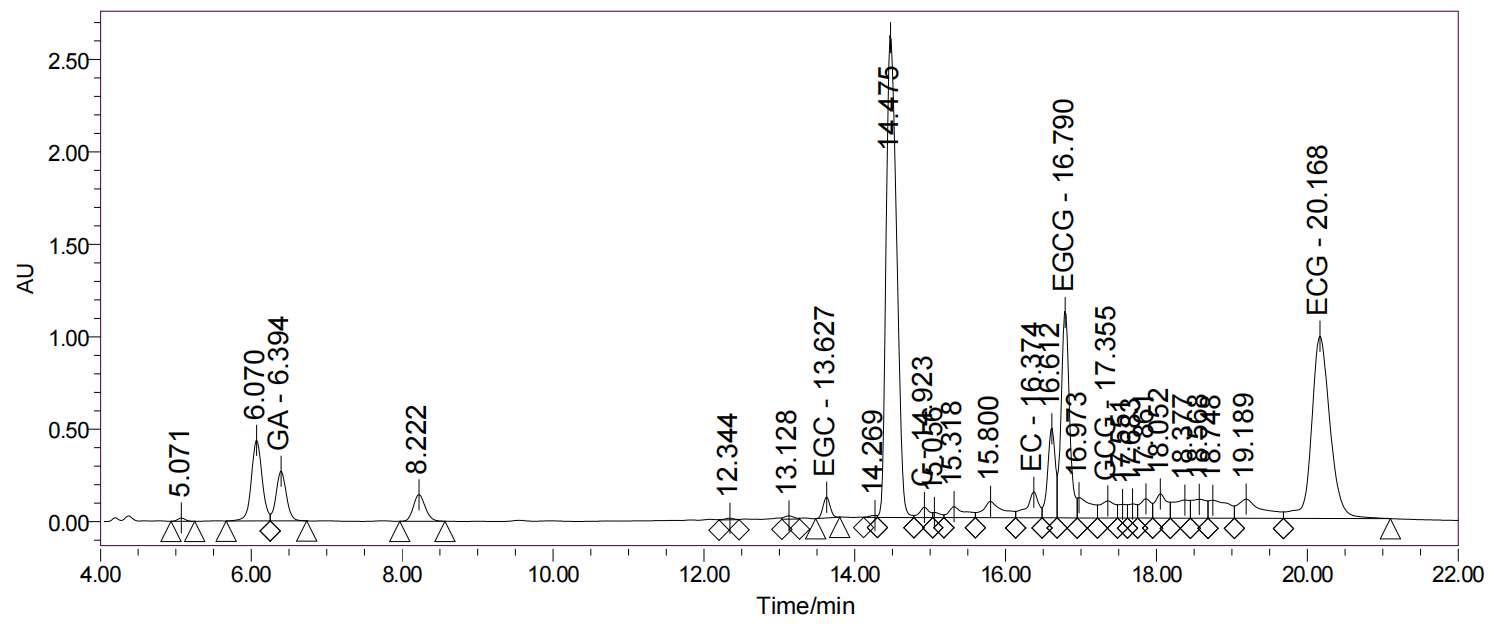


21QH-1


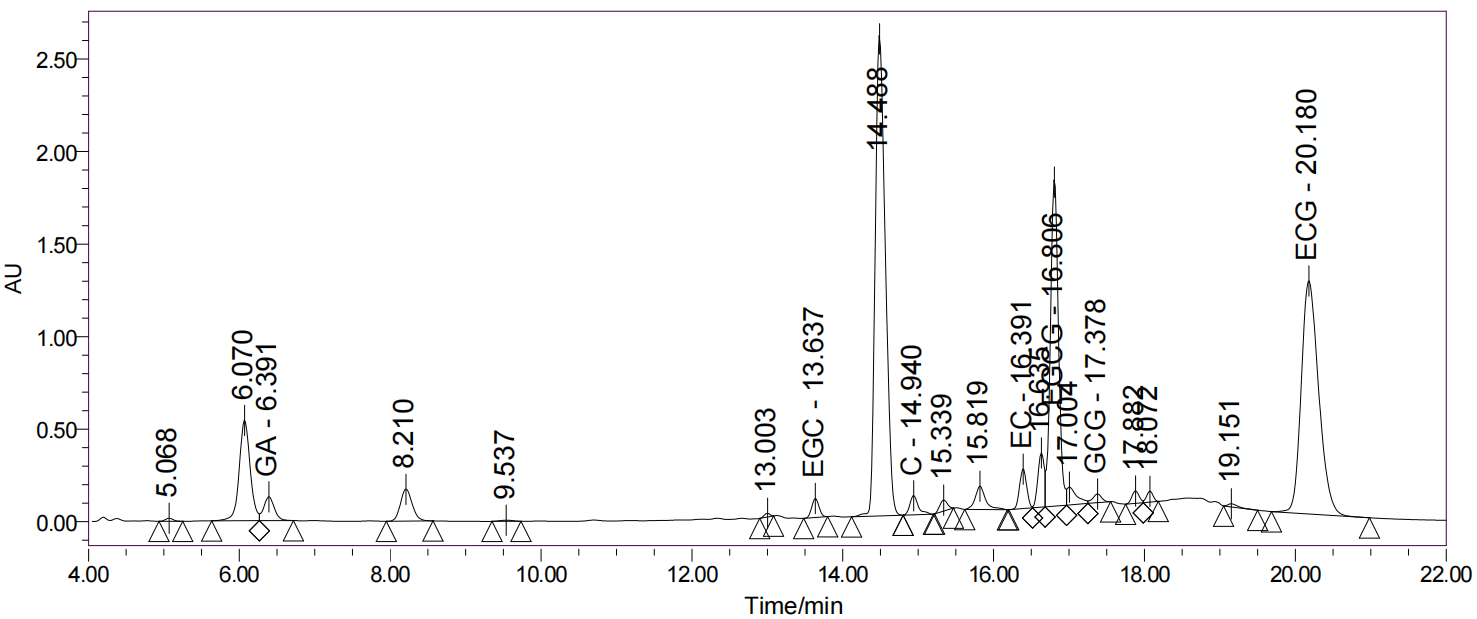


21QH-2


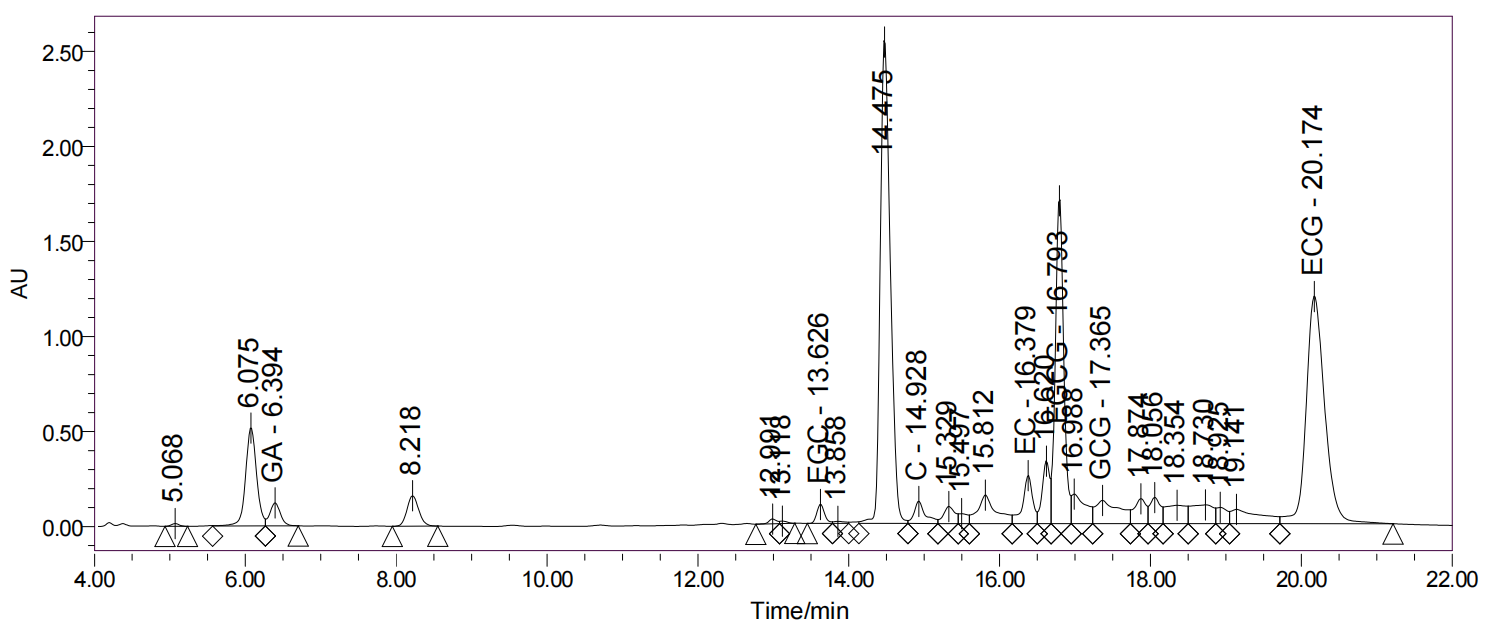


21QH-3


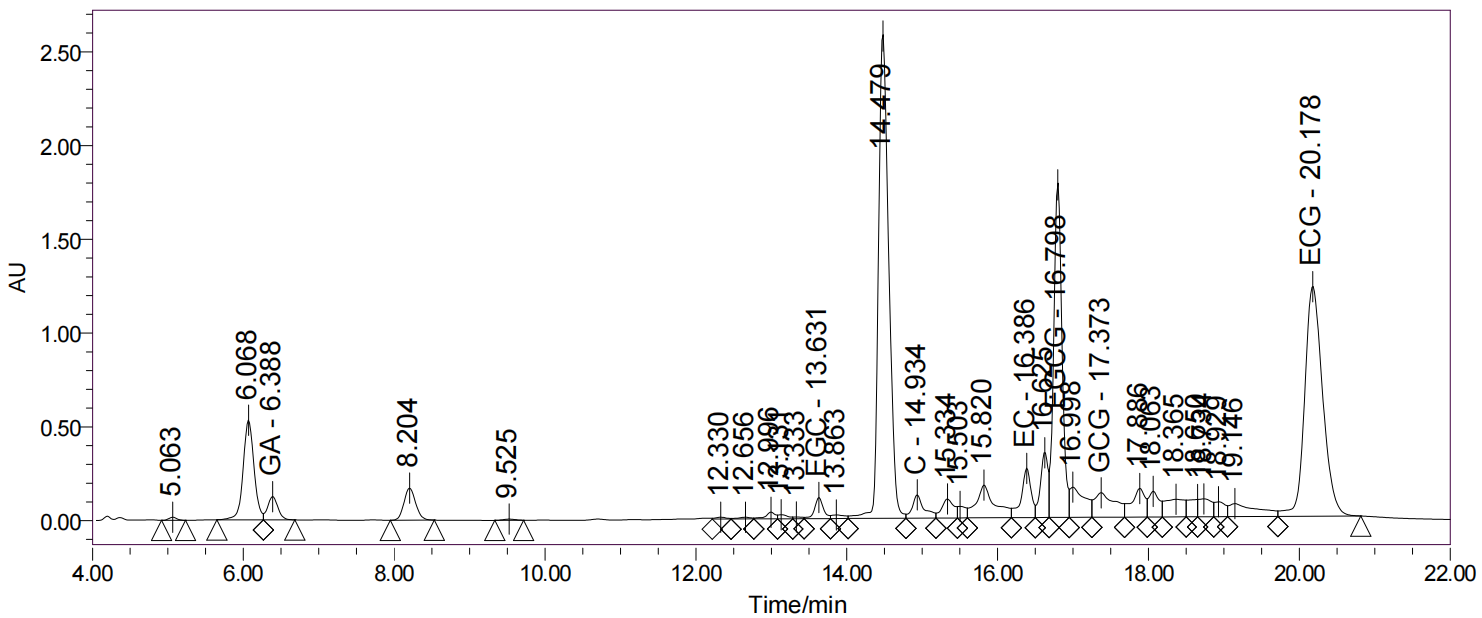


23QH-1


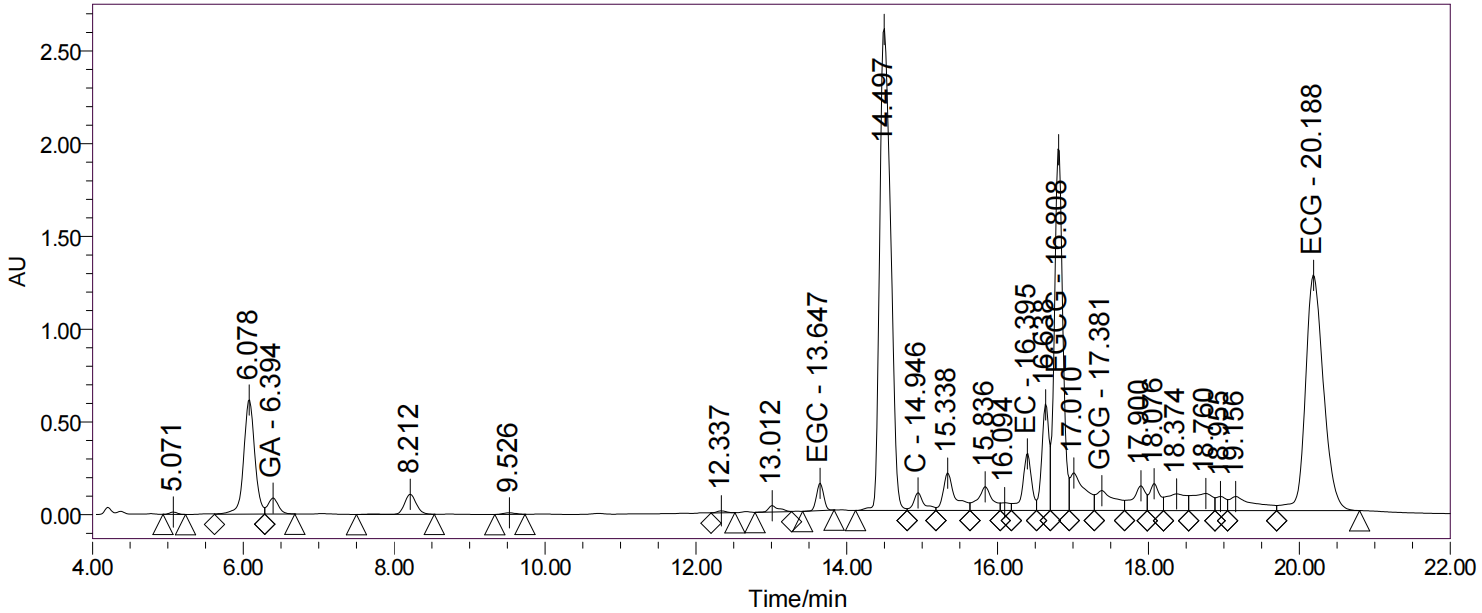


23QH-2


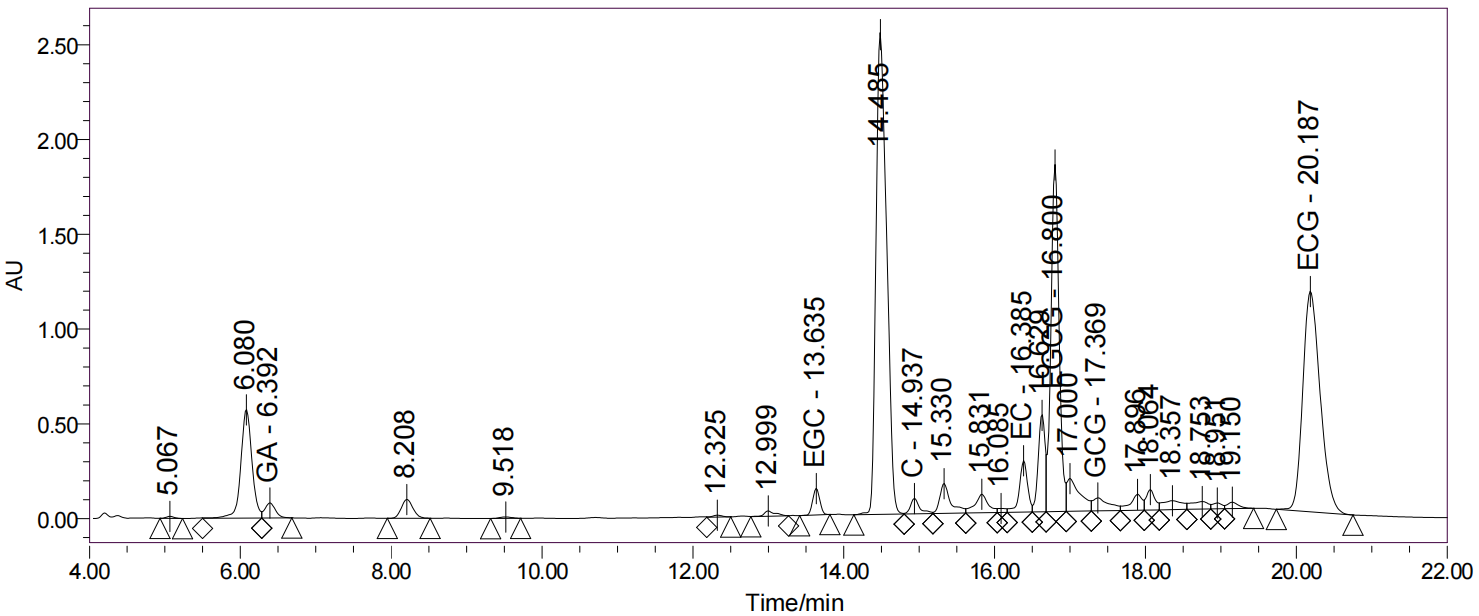


23QH-3


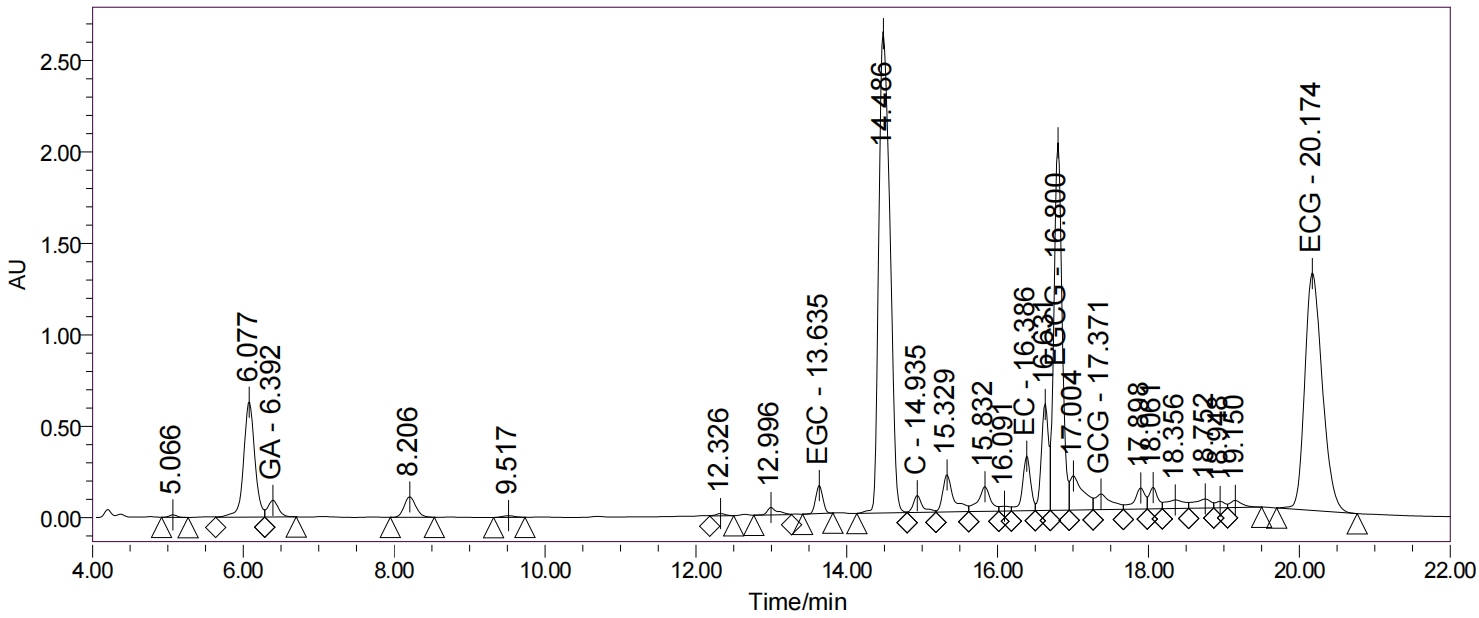


**Figure S2.** The chromatograms for catechins of QH teas in each sample.

AF-B1-CK


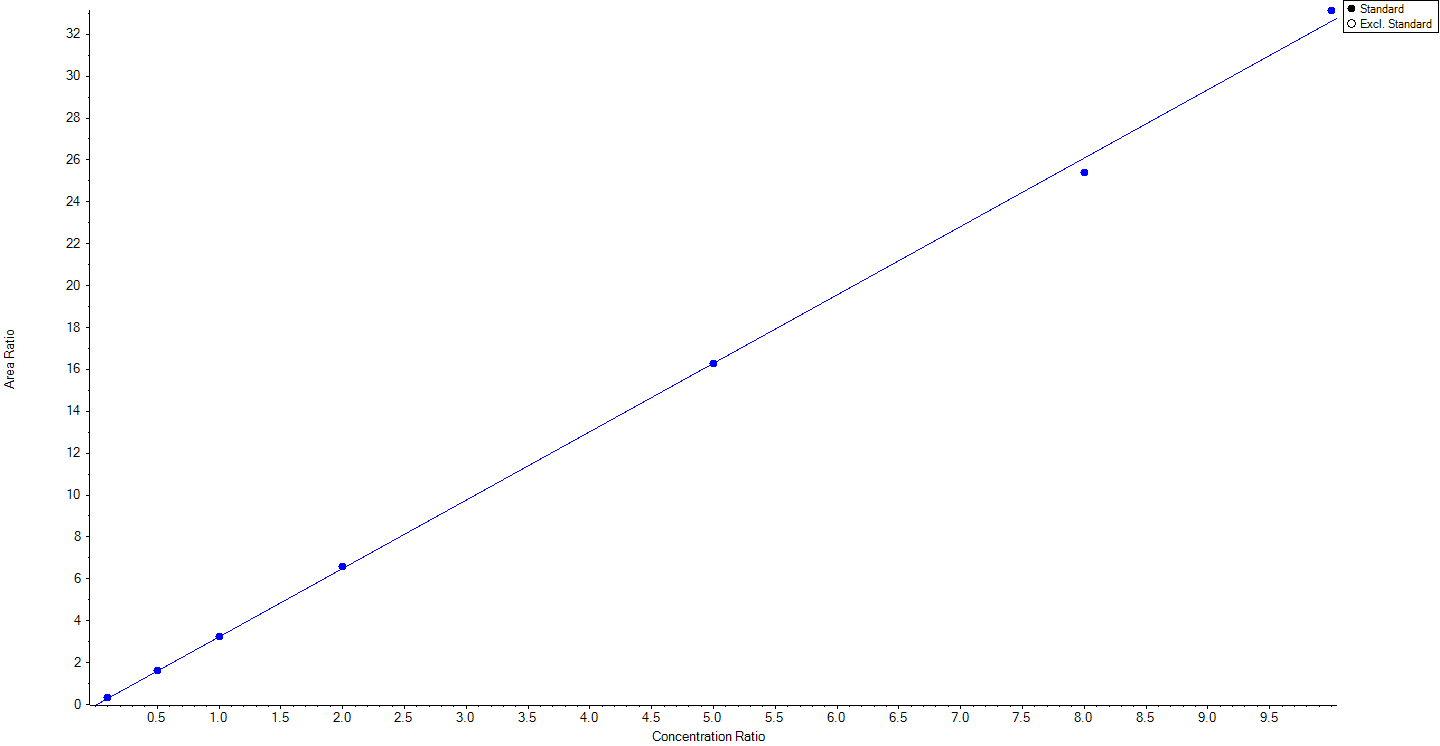


y = 3.26572 x + -0.03676 (r = 0.99964)

AF-B2-CK

y = 0.58078 x + 0.01502 (r = 0.99987)


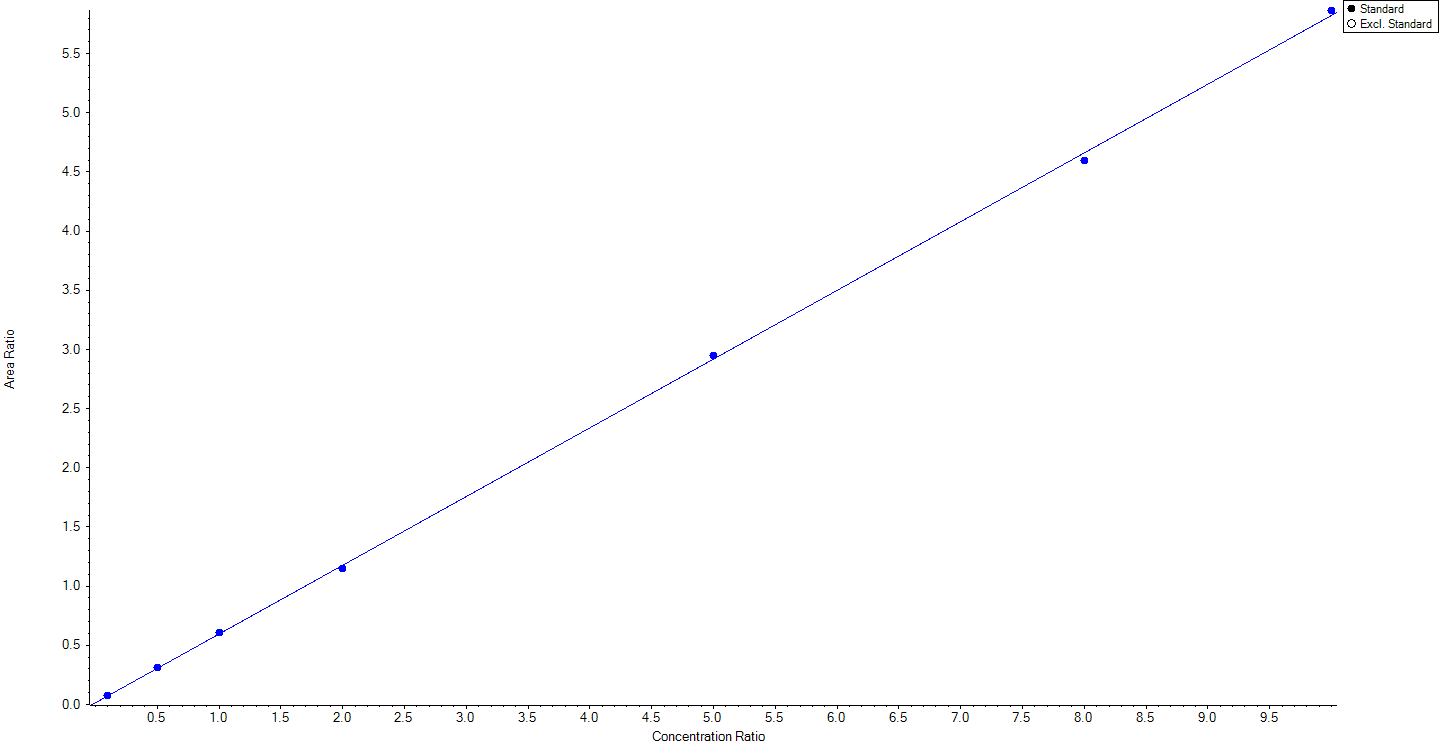


AF-B1-CK


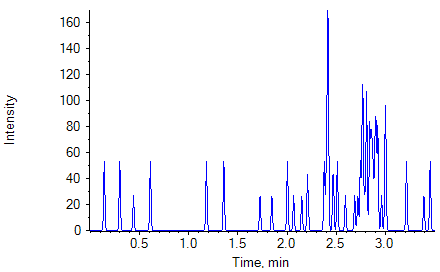

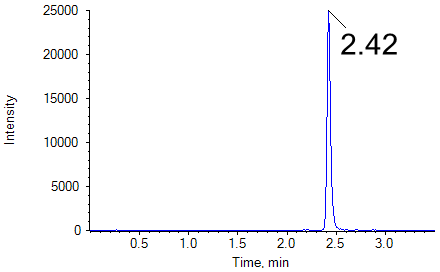


AF-B2-CK


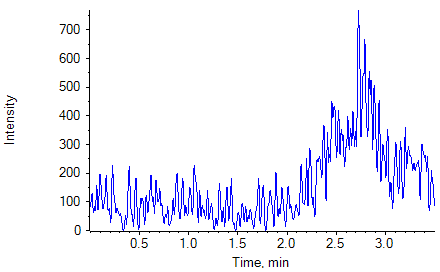

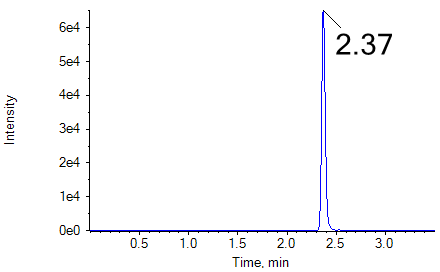


AF-G1-CK


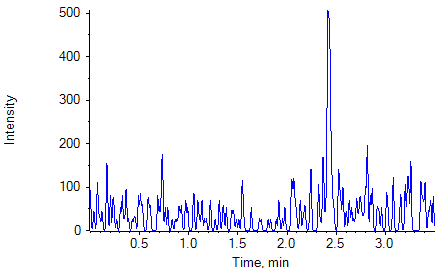

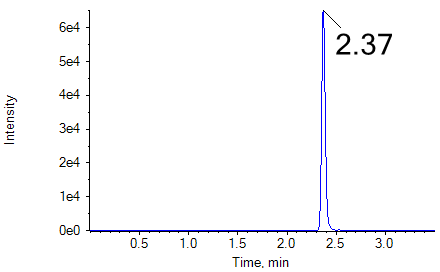


AF-G2-CK


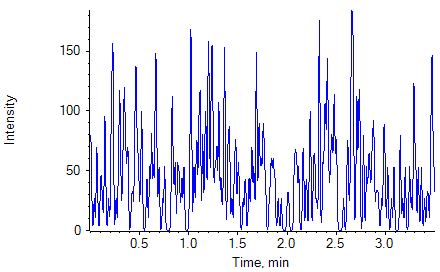

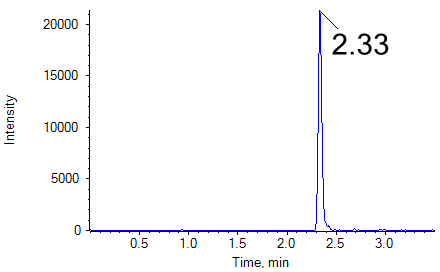


AF-B1-23QH


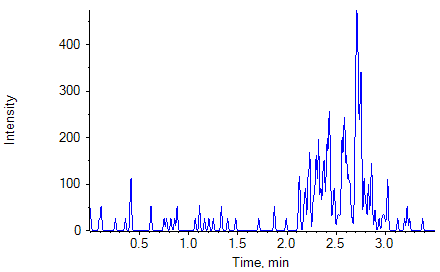

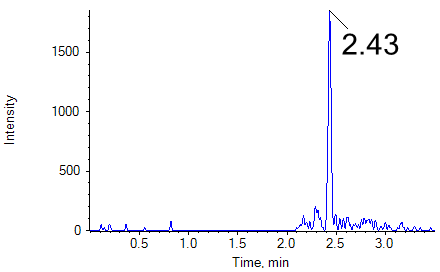


AF-B2-23QH


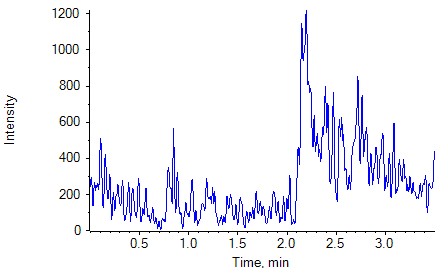

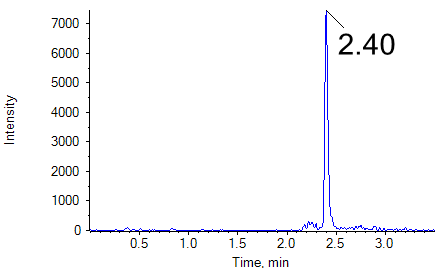


AF-G1-23QH


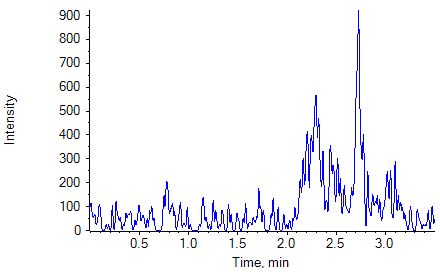

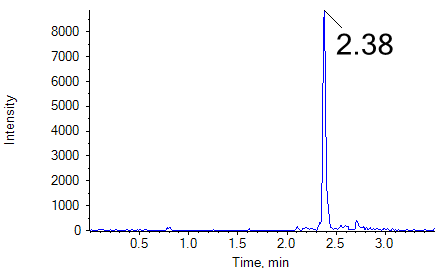


AF-G2-23QH


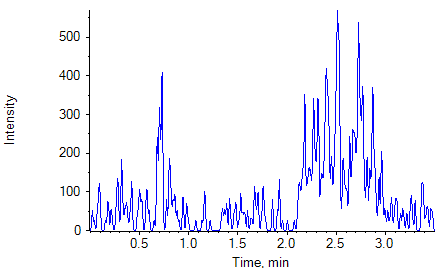

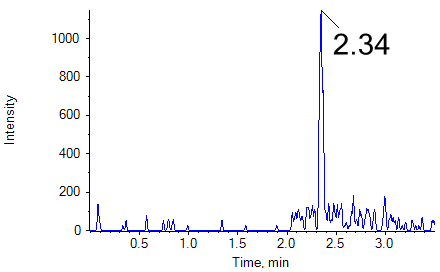


AF-B1-17QH


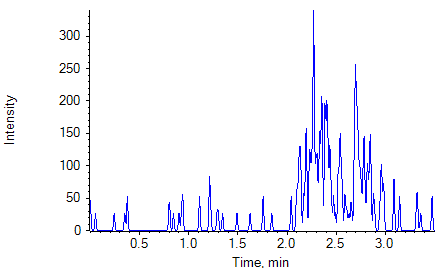

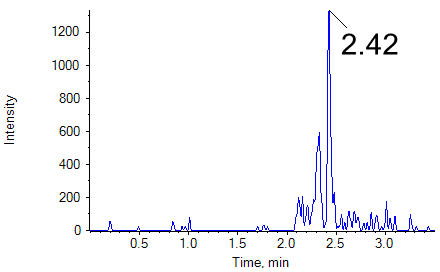


AF-B2-17QH


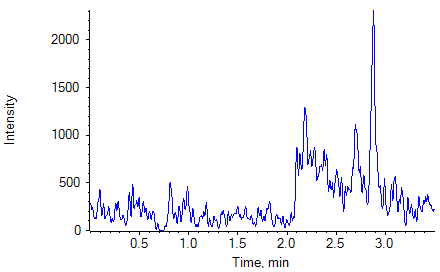

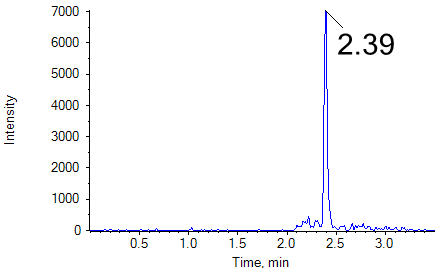


AF-G1-17QH


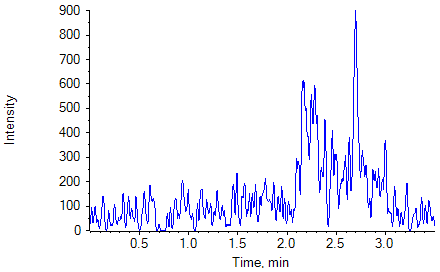

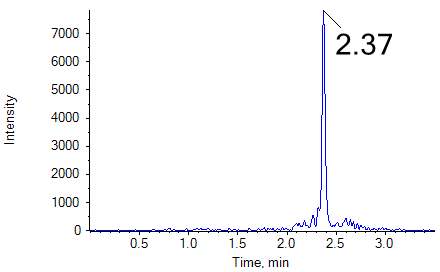


AF-G2-17QH


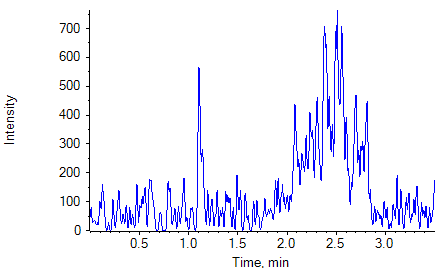

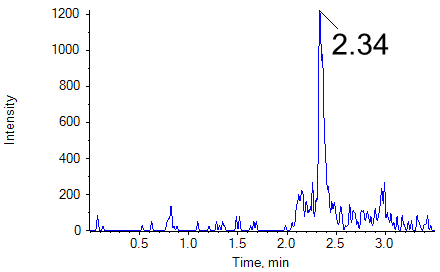


**Figure S3.** The standard curves and chromatograms for aflatoxins of QH teas in each sample.
